# Optimization of La_2_NiO_4+δ_ Electrolysis Cell Oxygen Electrode through Surfactant-Enabled LaCoO_3±δ_ Nanocatalyst Deposition

**DOI:** 10.1021/acsomega.5c08373

**Published:** 2025-10-29

**Authors:** Cole Klemstine, Javier Mena, Wenyuan Li, Awa Kalu, Xingbo Liu, Yu Zhong, Edward M. Sabolsky

**Affiliations:** † Department of Mechanical, Materials and Aerospace Engineering, 5631 West Virginia University, Morgantown, West Virginia 26506, United States; ‡ Department of Chemical and Biomedical Engineering, 5631 West Virginia University, Morgantown, West Virginia 26506, United States; § Department of Materials Science and Engineering, 8718 Worcester Polytechnic Institute, Worcester, Massachusetts 01609, United States

## Abstract

Lanthanum nickelate (LNO) has shown promise as a Cr-resistant air electrode material for SOECs but has suboptimal surface oxygen exchange properties. Nanocoating of the LNO surface with lanthanum cobaltite (LCO) was chosen to improve cell performance as a surface oxygen conductor. The work focused on the implementation of a two-step nano-LCO film deposition utilizing catechol molecules in a porous LNO electrode. The subgoals of the work were to maintain nanosized LCO particles/grains to increase active surface area and to control the regularity/homogeneity of the coating across the microstructure. To achieve these goals, a novel surfactant-enhanced liquid infiltration method was utilized, where nucleation sites were spread across the electrode structure to control the location and size of LCO particles. Various catechol surfactant compositions were evaluated for their ability to control the kinetics of nanoparticle deposition and the homogeneity of the coating. Chelated LCO was characterized by X-ray diffraction (XRD), which found a substantial improvement in LCO formation with surfactant addition and determined polymerized norepinephrine to be the best-performing surfactant, with 88.4% pure LCO formed at low temperature. X-ray photoelectron spectroscopy (XPS) confirmed LCO nanostructures formed by the two-step infiltration process, showing no impurities and a stable perovskite structure. Deposition kinetics were analyzed using atomic force microscopy (AFM), correlating infiltration times and solution molarity to nanoparticle size and distribution, the results of which were confirmed in symmetrical cell samples by scanning electron microscopy (SEM). Electrochemical impedance spectroscopy (EIS) testing demonstrated substantial improvements in polarization resistance, where the nanocoating reduced the resistance by ∼55% to 0.152 Ω·cm^2^ at 700 °C and 0.039 Ω·cm^2^ at 800 °C. Electrical conductivity relaxation (ECR) at this temperature confirmed an improved surface oxygen exchange coefficient of the LCO + LNO heterostructure predicted by the Bode data from EIS, alongside a reduction in activation energy by about 30%.

## Graphical abstract

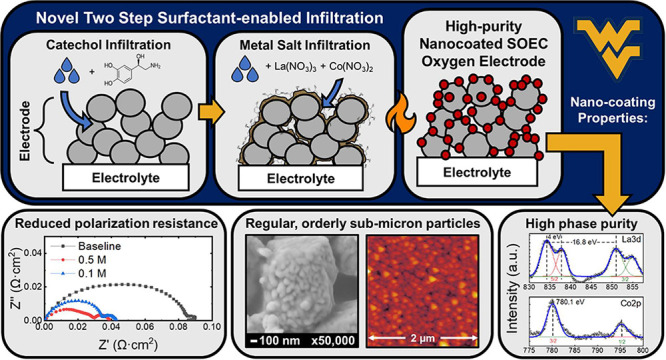

## 1 Introduction

As the world seeks to reduce its reliance on hydrocarbons, the demand for sustainable hydrogen production methods has become increasingly critical. The International Energy Agency (IEA) estimates that to meet the global energy demand sustainably, approximately 300 million tons of hydrogen production per year would be required by 2050.^1^ Solid oxide electrolysis cells (SOECs) represent a promising technology for hydrogen production within this context. SOECs perform high-temperature electrolysis, where steam (H_2_O) is reduced at the cathode into hydrogen (H_2_) and oxygen ions (O^2–^), which diffuse through the electrolyte to the oxygen electrode, where they recombine into oxygen (O_2_). Compared to other kinds of water-electrolyzers (e.g., alkaline electrolyzers, proton exchange membranes (PEM)), solid oxide electrolysis is much more efficient, having shown to have a power-to-fuel efficiency of 90% or greater.^2,3^ The technology is also flexible and able to process carbon dioxide and other mixtures containing gases like methane into cleaner equivalents. Solid oxide fuel cells (SOFCs) that process hydrogen to energy are well developed and optimized, but SOECs require more research in order to successfully implement and commercialize the technology.

The oxygen/air electrode was evaluated first based on the fuel-cell mode chemistries and then later on more SOEC-specific compositions. Some of the most prominent fuel cell materials that are viable candidates as oxygen/air electrodes in SOECs are La_0.6_Sr_0.4_Co_0.2_Fe_0.8_O_3–*x*_ (LSCF) and La_0.8_Sr_0.2_MnO_3–*x*_ (LSM). Chromium poisoning is a well-known problem for these compositions, where the presence of steam and the common use of stainless-steel interconnects result in Cr migration within the stack due to high-temperature gaseous chromium hydroxide formation. The high vapor pressure of chromium hydroxide enables it to react with many components within the cells, forming insulating solid phases, blocking active sites, and migrating to the electrode–electrolyte interface. This ultimately leads to delamination of the cells and long-term degradation of the system.^4^ LSCF was shown to be quite susceptible to chromium poisoning, as well as degradation to form Co_3_O_4_ and strontium segregation, leading to the insulating SrZrO_3_ phase.^5,6^ LSM also struggles with Cr-poisoning at the electrode–electrolyte interface, forming Cr_2_O_3_ and SrCrO_4_. These phases contribute to the delamination of the cell and mechanical weakening of the LSM microstructure due to intergranular precipitate formation and buildup of LSM nanoparticles at the electrolytic interface.^7,8^

Other electrode compositions of interest for SOEC applications are the Ruddlesden–Popper (R-P) structured phase. Examples most commonly include materials such as rare-earth-based nickelates like Nd_2_NiO_4+δ_ (NNO), Pr_2_NiO_4+δ_ (PNO), or La_2_NiO_4+δ_ (LNO). These perovskite-structured materials, containing no Sr, were found to have increased resiliency to Cr-poisoning,^9^ showing much less chromium deposition in the microstructure and fewer harmful phase formations. These compositions still struggle with the formation of rare-earth zirconate when in direct contact with doped zirconia electrolytes.^10^ Barrier layers composed of rare-earth-doped ceria, most commonly gadolinium-doped ceria (GDC), have shown success in isolating the zirconate formation and improving cell performance through high ionic conductivities. Additionally, R-P structured materials mixed with doped ceria were shown to further increase redox kinetics when utilized in SOFC mode, but were found to be reactive with low-doped ceria.^11,12^ This reactivity was found to be reducible both by increasing the rare-earth doping in the barrier layer near the solubility limit^13^ and by using a composite LNO:GDC at 50:50 wt %.^14^

With this updated understanding of A_2_NiB_4+δ_ materials and their interactivity with commonly used SOEC components, the reactivity was reduced to an acceptable range to minimize long-term degradation. LNO was proven to be compatible with common SOC electrolytes, such as GDC and YSZ, and showed high tolerance to chromium poisoning, making it a top candidate for effective, chromium-resistant SOECs. However, LNO struggles with a lower surface oxygen exchange, resulting in a higher area-specific resistance (ASR) when compared to LSCF.^15,16^ Thus, surface modification of this composition has become of interest to help improve the electrochemical performance of LNO.

It has been proposed to alter the surface chemistry of LNO with perovskite-structured lanthanum cobaltite (LCO) composition to improve charge transfer processes. The LCO composition was previously shown to display resistance to chromium poisoning over long-term testing in the presence of chromium.^17^ In order to achieve this nanocoating, many researchers over the past decade have focused on the use of an inexpensive and simple liquid infiltration process to deposit these nanocoatings within the porous electrodes. Liquid infiltration methods utilize nanoparticle dispersions, metal salts, molten salts, and surfactant-aided precursor systems. Aqueous metal salt solution infiltration is the most common method, which utilizes single-component metal salts dissolved in a water-based solvent, which is pipetted or sprayed into the porous electrode microstructure. Upon drying, metal hydroxide or oxide nanoprecipitates are formed through the porous microstructure, and the homogeneity, percolation, and film thickness will depend upon various solution additives, concentrations, and drying conditions. Typically, this method has been demonstrated for the enhancement of both anode and cathode SOFC electrodes using precursors that produce single-component metals like Pd, Pt, and Ag^18–20^ and metal oxides such as CeO_2_ and CoO.^19,21,22^ Additionally, these methods struggle with the deposition of complex oxides due to solubility differences between ions, causing segregation during the final drying phases of the infiltration. For more complex chemistries, a chelating agent is necessary that has equal affinity for multiple ion types with differing solubilities. This aids in the control of both the placement of nanocoating across the cell and the precipitation of the desired ions in a chemically homogeneous order. Prior research showed that the chelation of nanocoatings using catechol molecules is an effective and reliable solution to allow for precise decoration throughout the cell that only requires a single infiltration step to reach adequate loading.^23^

In this work, the liquid solution impregnation of the ternary oxide LaCoO_3_ (LCO) was studied as a potential method for nanocoating the La_2_NiO_4+δ_ composition to improve its surface oxygen exchange for potential use as an SOEC anode. In order to control precipitation and nanoparticle formation, a specialized surfactant was used within the porous structure as a chelating agent. This work focused on the use of catechol or catechol-like molecules, including polynorepinephrine (pNE), caffeic acid, dihydroxybenzoic acid (DHBA), and gallic acid. Previous work from Ozmen et al. and Wang et al. showed the success of bioinspired catechols, such as pNE, caffeic acid, and DOPA, to deposit single-component nanoparticles^23,24^ onto planar and some porous microstructures. This work expands on their work to include both new catechol-family molecules as chelating agents and to attempt deposition of more complex ternary oxides never demonstrated before. A successful chelating agent will serve three purposes: (1) act as a complexing agent in the formation of complex, ternary oxides, (2) assist in the deposition of these chemistries in distinct, nanorange structures, and (3) assist in the control of the homogeneity of these coatings, with optimal coverage statistics.

The work will determine the most suitable candidate for the chelation of the La and Co oxides and/or hydroxides initially, enabling LCO perovskite phase formation at temperatures below 900 °C. This low temperature was chosen to allow LCO particles to remain nanosized. The complexing properties of the chosen chelating agents were analyzed by initially analyzing the LCO phase formation within precipitation studies using X-ray diffractometry (XRD). Nucleation and growth characteristics of LCO coatings were then studied on single-crystal YSZ using atomic force microscopy (AFM) to determine the impact of deposition parameters on the loading rate of the samples and the final microstructure of the nanocoatings. Additionally, these samples were evaluated using X-ray photoelectron spectroscopy (XPS) for a second level of validation for the results of phase-purity testing by analysis of binding energies. Results from these experiments were implemented on the LNO – LNO/GDC composite porous electrode microstructures to determine their impact on the performance of the coated cell. The microstructures of the infiltrated LNO microstructures were characterized using scanning electron microscopy (SEM). Finally, the polarization resistance of the LCO-coated LNO was evaluated by symmetrical cell tests using electrochemical impedance spectroscopy (EIS), and the surface exchange rates were measured using electrical conductivity relaxation (ECR).

## 2 Experimental Section

### 2.1 Synthesis of Materials

#### 2.1.1 Ex Situ Synthesis of LCO Powder Using Catechol Surfactants as Chelating Agents

To determine the capability of the chosen surfactants to deposit high-phase purity, ternary LCO, an ex situ precipitation and phase development study was conducted. La­(NO_3_)_3_·6H_2_O (99.9%) and Co­(NO_3_)_2_·6H_2_O (>98%, Thermo Fisher, Waltham, MA, USA) as metals source were individually mixed with 50/50 by weight ethanol–water to create lanthanum and cobalt molar solutions of 0.5 M. Molarity of the solutions was confirmed by trace-ion testing using an inductively coupled plasma-mass spectrometer (ICP-MS PerkinElmer NexION 2000, USA) following the EPA Method 200.8. The resultant 0.5 M solutions were then combined with each surfactant at a chosen molar concentration. Figure 1 shows the four molecules chosen as chelating agents during testing; norepinephrine (NE, >97%, Sigma-Aldrich, St. Louis, MO, USA), caffeic acid (>98%, Tokyo Chemical Industry, Tokyo, JPN), gallic acid (97%, Alfa Aesar, Ward Hill, MA, USA), and DHBA (99%, Alfa Aesar, Ward Hill, MA, USA). All were chosen because of the complexing properties associated with the catechol molecular family. The proposed approach assumes that different chelating agents will enable hydroxyl groups with different spacings, which can incorporate alternative ion populations and result in the optimal atomic spacing to form the desired LCO phase. The solutions were dried to form a gel and heated to 300 °C to decompose and oxidize the nitrates. Resultant powders were ground using a mortar and pestle to reduce powder size and heat-treated to 600, 700, and 800 °C. Final powders were ground again by mortar and pestle, and then analyzed using XRD to determine crystal structure and complete phase development analysis.

**Figure 1:**
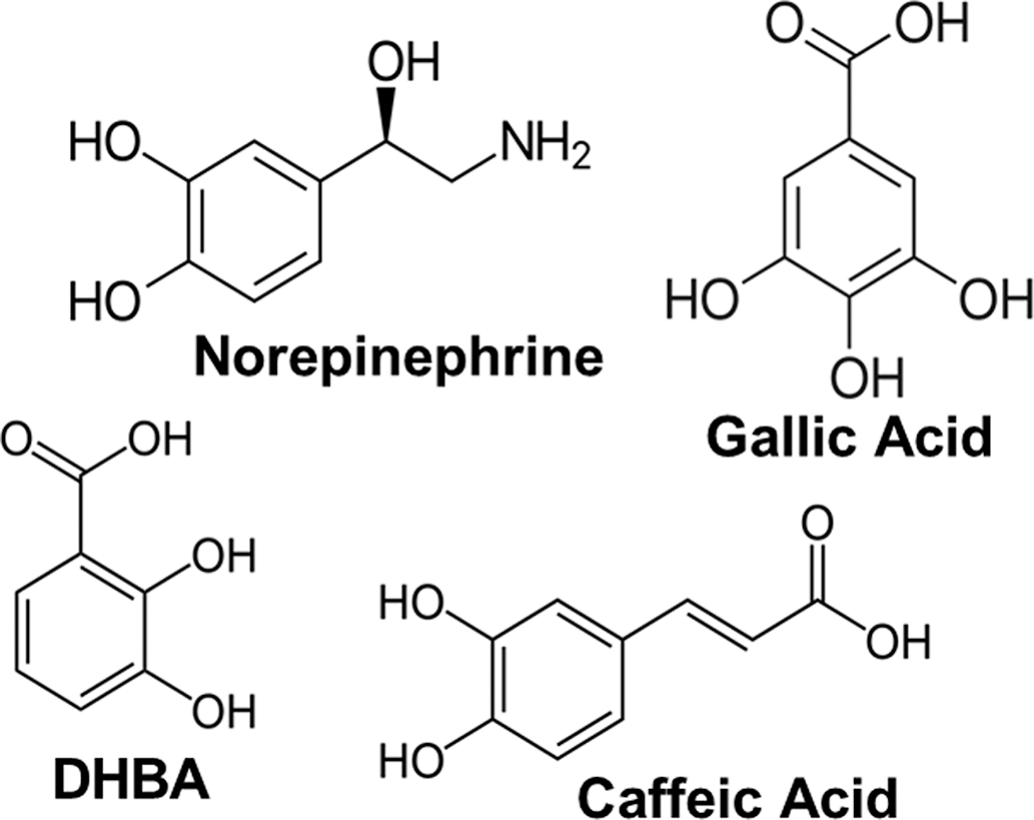
Selected catechol-family molecules used as potential surfactants to chelate LCO.

#### 2.1.2 XRD of Ex Situ LCO Powders

Phase purity of prepared LCO powders was examined by X-ray diffraction (XRD, PANalytical X-pert PRO, Cu Kα radiation, Model PW 3040 Pro). Operating parameters for power were 45 kV and 40 mA. The X-ray beam divergence slit angle was set to 0.5°. Scans were taken at a scan rate of 0.0711°/s and a 29.85 s step time. X’Pert HighScore Plus software was used for analysis of phase structure and crystal structure, as well as Rietveld method analysis to determine quantitative phase composition. The same instrumental settings were used for the prepared LNO powder phase analysis. This testing determined that pNE was the optimal chelating agent, so it should be noted that for future experimental sections, only pNE will be used.

#### 2.1.3 Synthesis of LNO Powder

Lanthanum nickelate was synthesized using a solid-state reaction where La­(CO_3_)_2_·6H_2_O (99.9% purity) and NiO (78.5% purity) (Thermo Fisher, Waltham, MA, USA) as metal sources were combined with isopropanol, followed by attrition milling using a Szegvari Attritor System (Union Process, Akron, OH, USA) for 4 h. This mixture was then dried, ground with a mortar and pestle, and sifted using a 60 mesh (250 μm particle size) to reduce and homogenize the particle size before sintering. The final powder was fired to 1200 °C for 4 h, reground, and sieved through a 60 mesh to ensure particle size uniformity.

### 2.2 Sample Preparation and Characterization Methodologies

#### 2.2.1 LCO Deposition on YSZ Single-Crystal Substrates

LCO nanofilms were deposited on the surface of polished YSZ single-crystal substrates to allow both characterization of the LCO phase when chelated as a nanofilm, and of the deposition characteristics such as uniformity of coating and particle size on the final nanocoating. The variables studied were oxide precursor solution molarities, surfactant concentration, and deposition times for both surfactant treatment and precursor salt solution deposition. For each sample, a 50–50 by weight ethanol–water solution was combined with pNE at varying concentrations and tris­(hydroxymethylaminomethane) (TRIS) to act as a pH buffer, maintaining the solution at 8.5. Single-crystal polished YSZ substrates (100) orientation (MTI Corporation, Richmond, CA, USA) were placed in the solution and agitated by using a rocker plate for 24 h to promote polymerization. The substrates were then placed in various molarity LCO salt solutions to chelate into an LCO nanofilm across the surface. A second set of samples was prepared where, after pNE infiltration, substrates were gently rinsed to reduce the amount of active chelation sites for LCO. All final samples were fired to 800 °C as determined by phase-purity studies.

#### 2.2.2 AFM and XPS Analysis of LCO on YSZ Single-Crystal Substrates

Prepared LCO-coated YSZ substrates were analyzed using noncontact AFM (Asylum MFP-3D, Oxford Instruments, Abingdon, Oxfordshire, UK). AFM tips were tuned using the Stable Asylum Research programs' autotuning feature, and the laser was aligned. The set point was tuned alongside drive amplitude and integral gain until the highest image fidelity was achieved. Locations of 20 × 20 μm^2^scanned with 256 × 256 pixels were taken at a scan rate of 1.0 Hz, and then refined to 2 × 2 μm^2^, scanned with 512 × 512 pixels areas at a scan rate of 1.0 Hz, showing the most typical coverage of the cell. Images were postprocessed with Gwyddion 2.61 for particle height, sample roughness, and kurtosis. XPS analysis (PHI 5000 VersaProbe Photoelectron Spectrometer, ULVAC-PHI, Chigasaki, Kanagawa, Japan) was completed on the same LCO-coated YSZ substrates to determine binding energies of La and Co when deposited in a nanofilm and to detect potential additional unbonded chemistries. Peaks were deconvoluted using a Tougaard background and then curve fit to determine exact binding energies.

#### 2.2.3 Ink Synthesis and Heterostructured LNO-LNO/GDC Symmetrical Cell Printing

Electrolyte-supported button symmetrical cells were used in this study. Each cell was manufactured with custom-printed materials. Each cell consisted of a 20 mm diameter, 250 μm thick YSZ electrolyte (Product 211102, Fuelcellmaterials.com), a 12.5 mm diameter, 4 μm thick 20% Gd-doped CeO_2_ (GDC) layer, a 13 μm thick mixed LNO/GDC interlayer (12.5 mm diameter), and a 50 μm thick LNO bulk layer (12.5 mm diameter). The LNO mixed interlayer was used to increase bonding between layers due to differences in particle size. To print these layers, three inks were synthesized. A GDC barrier layer ink was created using GDC. A 0.1–0.4 μm GDC powder was mixed with 5–10 nm powder in an 80–20 ratio (Products 113102 and 111102, Fuelcellmaterials.com), then combined with ethanol, fish oil, and milling media. The mixture was attrition-milled and dried to a higher viscosity at around 60 °C, upon which the final slurry was combined with an ink vehicle (Batch T031P7, Johnson-Matthey Inc., Smithfield, PA, USA). An LNO bulk layer ink was made with synthesized LNO powder combined with the organic ink vehicle and fish oil, and sonicated until the proper printing viscosity was reached. A third 50/50 LNO-GDC mixed interlayer ink was created with 0.1–0.4 μm GDC and synthesized LNO measured to equal weights and combined with ink vehicle and fish oil. All inks were sonicated by using a sonic wand (Sonics VCX-130 Vibra-Cell). Using an Aremco Accu-Coat 3230 screen printer with a 325 mesh, 45.0° angle screen from UTZ Technologies, a layer of GDC was printed onto YSZ electrolyte and sintered to 1425 °C in a clean muffle furnace to ensure a dense and nonporous barrier layer. On the sintered GDC layer, a single layer of 50/50 LNO-GDC ink was printed with a 325 mesh, 45.0° angle screen and dried, upon which two layers of LNO-only ink were printed with a similar screen to ensure a large bulk area. Completed cells were heated in a tube furnace (Nabertherm RHTH 120/150/18, Lienthal, Germany) at 1 °C/min to 600 °C and held for 1 h to allow for binder burnout, and then heated at 2 °C/min to 950 °C and held for 2 h for final sintering. Cell porosity was measured by BET surface area analysis by Particle Technology Laboratories using a TriStar II 3020 (Micromeritics, Norcross, GA, USA). These symmetrical cells were used as the baseline cells for the nanofilm impregnation studies.

#### 2.2.4 LCO Infiltration into LNO-LNO/GDC Symmetrical Cells

Deposition of an LCO nanofilm was completed utilizing the surfactant-enhanced deposition process using polymerized norepinephrine (pNE), which was determined to be the best chelating agent in the powder study (further discussed in the Results and Discussion section). Figure 2 depicts a diagram of the surfactant layer growth process. In this process, a surfactant layer is initially deposited across the inner surfaces of the cell, allowing control of the nucleation and growth characteristics of the desired nanofilm. The chosen catechol molecule is suspended in an aqueous solution and polymerized to form a reactive mesh. The salt solution is impregnated into the newly formed molecular mesh, where ions are chelated into metal hydroxides by the active OH^–^ groups of the polymer chain. Finally, the nucleation sites further grow across the surface to form a well-distributed nanoparticle or nanofilm coating, depending on the coverage characteristics of the polymer. These can be adjusted via tuning of the surfactant concentration and sample immersion time. For infiltration of symmetrical cells, pNE was measured to a concentration of 2 mg/mL and mixed with a solution of 50/50 ethanol–water to reduce surface tension and further enable capillary action. Once mixed, tris-hydroxymethyl aminomethane (TRIS, 99%, A18494, Alfa Aesar, Ward Hill, MA, USA) was added at a concentration of 0.05 M to raise the solution pH to 8.5, acting as a buffer and beginning molecular polymerization. Symmetrical LNO cells were submerged in a 50 mL beaker of the pNE catechol/TRIS solution. Cells were held vertically in solution by a plastic spacer and placed in a vacuum chamber under a vacuum of 30 mmHg for 30 min to aid in capillary action and to remove air inside cells. This allowed the surfactant solution to fully saturate and penetrate the porosity of the LNO to the active layer, where nanoparticle coverage needs to exist. After saturation, cells were agitated on a rocking platform shaker at ∼0.5 rpm for 24 h to ensure total coating of internal surfaces. After the initial coating process, the cells were then placed in DI water to gently rinse and remove unbonded molecules. Lanthanum nitrate and cobalt nitrate precursor solutions were mixed to form a 0.5 M mixed lanthanum and cobalt nitrate solution. The surfactant-treated symmetrical cells were submerged in these solutions and vacuumed to remove air and pull ionic salt solutions into the microstructure. Infiltrated cells were agitated using the shaker at ∼0.5 rpm for 6, 12, and 24 h time periods to allow for different levels of LCO nanofilm growth to occur. After film deposition, the cells were gently rinsed in the beaker of DI water to remove unbonded nitrates on the cell surface and then fired in a tube furnace to 600 °C at 1 °C/min, held for 1 h for nitrate decomposition and carbon removal, and then to 800 °C at 2 °C/min and held for 2 h for sintering. The chosen temperature was lower than the usual sintering temperature for these materials, since the goal is to simply bond the particles to the backbone and transform the chemistry into the desired phase while maintaining the nanostructure of the film. Due to possible small variations as a result of the infiltration method yielding mass gains in the 10^–5^ range, representative YSZ pellets were used for nanocoating loading calculations. Pellets were pressed with varying surface areas, measured by BET from Particle Tech Laboratories (Downers Grove, IL), and then infiltrated at various conditions. This allowed an increase in the magnitude of the LCO mass for measurement and analysis of the effect of the infiltrated sample surface area on loading. Loading rate for 12 h infiltrations was estimated by this method to be ∼1.5 and ∼2.0 mg/m^2^ for 24 h infiltrations.

**Figure 2:**
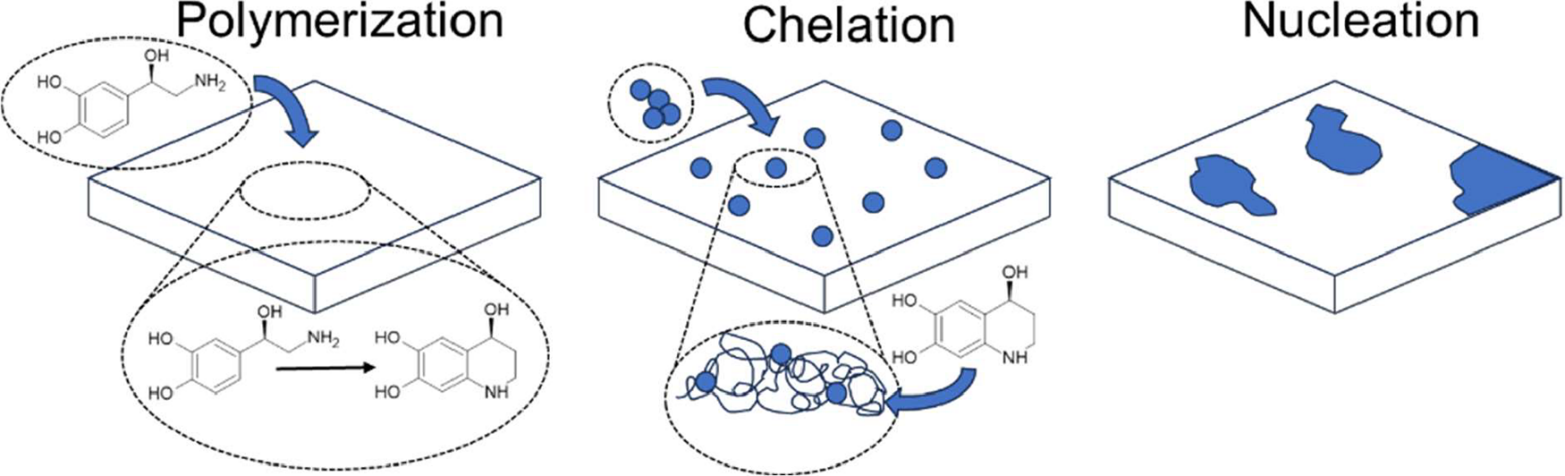
Steps involved in the surfactant layer growth process.

#### 2.2.5 SEM Analysis of Baseline and LCO-Coated LNO-LNO/GDC Symmetrical Cells

Cells were analyzed both pre- and post-mortem following electrochemical testing. Prior to SEM, cells were edge-cracked to provide a cross-sectional sample, then sputtered (Au sputtering target, Denton Desk V, Moorestown, NJ, USA) at 18 mA for 120 s. All SEM images were acquired using a JEOL JSM 7600F. Images were taken at 5 kV to prevent charging and allow for higher image quality.

#### 2.2.6 EIS Characterization of Baseline and LCO-Coated LNO-LNO/GDC Symmetrical Cells

To prepare the samples for EIS, a silver current collector mesh with leads was attached to symmetrical cells using LNO-percolated silver paste. Silver ink was prepared with the same ink vehicle as that discussed above. Two particle sizes of silver (0.7–1.3 and 4–7 μm) were percolated with 10% doping of LNO. This was used to enhance adhesion and minimize shrinkage of the Ag layer that may lead to delamination of the cell or electrode across the characterization process. After the current collector layers and leads were attached, the cell was heated to 600 °C for 60 min to achieve thermal equilibrium and allow sufficient time for binder burnout. Cells were measured from 600 to 800 °C in 50 °C increments. EIS was completed at each of these temperatures to obtain polarization resistance curves. A current of 0.01 A rms and a scanning range from 1 MHz to 0.05 Hz were used. Cells were thermally equilibrated for 30 min at each tested temperature to ensure standardized results. All measurements were completed by utilizing a Gamry Reference 600 system (Gamry Instruments, Warminster, PA, USA).

#### 2.2.7 LNO Bar Sample Preparation and LCO Coating/Infiltration

Bars made of the cell backbone material, LNO, were pressed to allow for oxygen exchange coefficient testing via ECR. LNO powder was created using a mixture of La­(NO_3_)_3_·6H_2_O (99.9%, Thermo Fisher, Waltham, MA, USA) and Ni­(NO_3_)_2_·6H_2_O (Sigma-Aldrich, St. Louis, MO, USA), and phase purity was confirmed using XRD. 1.5 g LNO powders were dry pressed into pellets in a 15 mm diameter die and then densified at 1200 °C for 4 h. The round pellets were cut by a diamond saw (Isomet Low-Speed Saw, Buehler, Lake Bluff, IL, USA) into bar samples with dimensions of 1 × 3 × 15 mm^3^. A slurry of LCO perovskite was prepared using the same lanthanum nitrate batch and Co­(NO_3_)_2_ · 6H_2_O (98%, Sigma-Aldrich, St. Louis, MO, USA) to coat the surface of the LNO bar samples. Five grams of phase-pure powder were attrition-milled (20T Automatic Lab Press, MSE Supplies, Tucson, AZ, USA) in ethanol for 6 h. Upon completion of the milling process, the slurry was collected and dried thoroughly at 100 °C for 2 h. Two grams of powder were weighed and mixed with 2.4 g of ink (4% ink VEH) prepared using a solvent of α-terpinol (10482-56-1, Sigma-Aldrich, MO, USA) and a solute of ethyl-cellulose (9004-57-3, Thermo Fisher, Waltham, MA, USA). The prepared slurry was coated on several samples and then dried. A second sample was prepared where LCO nanoparticles were infiltrated across the surface of the bar, simulating the nanocoating completed for symmetrical cells. Deposition parameters for this infiltration were a standard pNE deposition time of 24 h at a concentration of 0.1 M and an LCO deposition time of 12 h at a concentration of 0.5 M, as determined by the most successful symmetrical cell samples. All coated bar samples were sintered to a temperature of 800 °C before being prepared for the ECR measurement. LNO dense bar sample without any coating is denoted as LNO. LNO bars coated with a full, phase-pure LCO slurry coating are denoted LNO + full LCO, and those with a nanoparticle LCO surface coating are LNO + nano-LCO. This process was repeated with samples left in a disc shape to better observe trends occurring in larger sample sizes.

#### 2.2.8 ECR Characterization of Baseline and LCO-Coated LNO Bar Samples

All bars and disc pellets were prepared for ECR testing. Each side of the samples was connected to two silver wires (four total) using silver paste, and the samples were sintered to 500 °C for 2 h to enable a strong connection between the lead wires and the sample surface. Samples were measured by the 4-probe ECR method, utilizing a 6.5-digit multimeter (Keysight, Santa Rosa, CA, USA) for voltage measurements, utilizing a current source (Keithley Instruments, Cleveland, OH, USA) outputting 0.2 A. A mass flow controller (Alicat Scientific, Tucson, AZ, USA) was used to regulate the gas content of the 20:80 oxygen–nitrogen mixture at a flow rate of 400 sccm. For every sample, a range of PO_2_ was tested, starting at 0.2 atm and finishing at 1.0 atm, to understand the behavior at different potential electrolysis conditions. Bar samples were tested at an operating temperature of 700 °C, while disc pellet samples were tested at an operating range of 500 to 700 °C at 100 ° intervals.

## 3 Results and Discussion

### 3.1 LCO Phase Characterization via Powder Study

Before initiating in situ studies on symmetrical cells, precipitation studies of bulk LCO powder as a function of catechol-based chelating agent were initially evaluated, allowing the prescreening of the catechol-based chelating agents on the phase formation of the desired ternary composition at low temperatures. Low temperatures (600–800 °C) were necessary to prevent overdensification and grain growth of the nanoparticles within the target LNO backbone microstructure. Final powder samples were analyzed with XRD to determine powder composition and quantify phase formation percentages by utilization of the Rietveld analysis. Variables evaluated for these samples were the calcining temperature, surfactant type, and surfactant concentration. Figure 3 shows the XRD spectra of the pNE-chelated LCO powder across a range of calcination temperatures. For this figure, the pNE concentration was kept constant at 0.01 M. The XRD spectra show a decrease in the intensity of secondary phases as the heat treatment temperature increases. At 700 °C, La_2_O_3_ peaks of the {100}, {002}, and {101} planes in the 25–30° region dominate. These peaks receded to lower intensities at 800 °C calcination temperature. CoO peaks also decrease in amplitude, correlating with heat treatment temperature. As these peaks recede, the intensity of LaCoO_3_ peaks increases, indicating that higher calcination temperature aided in the formation of LCO perovskite. This analysis process was repeated for the three chelating agents. The XRD spectra were analyzed using Rietveld analysis to determine phase percentages, the results of which are depicted in Figure 4. As can be seen, the use of pNE as a chelating agent resulted in the highest chelated LCO phase percentage, where 49 and 61.3% transformation was achieved at 700 and 800 °C, respectively. Additionally, the increase in temperature resulted in a higher formation of LCO and a decrease of La and Co oxide impurities across all samples, as was previously observed in the spectra of Figure 3.

**Figure 3:**
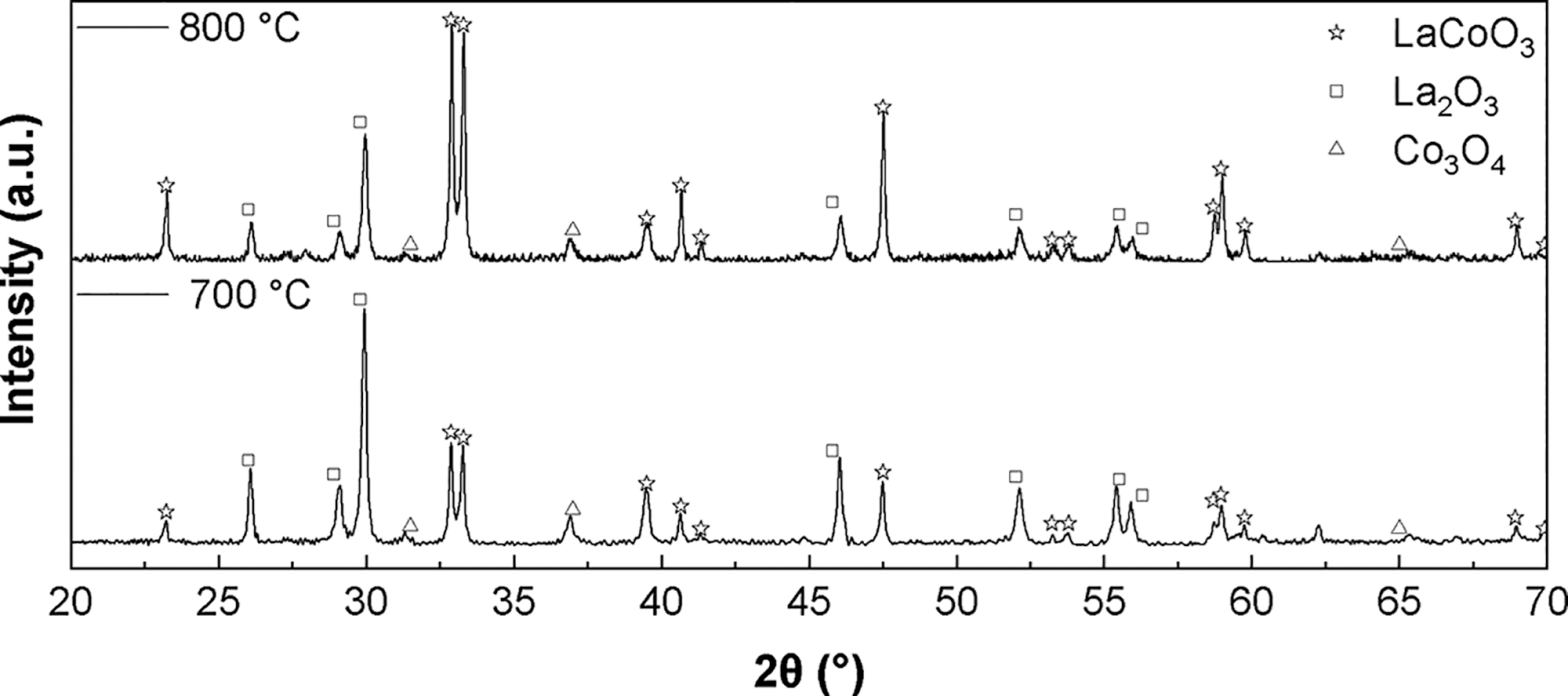
XRD diffractrogram of LCO powder chelated with pNE at a concentration of 0.01 M and calcination temperatures of 700 and 800 °C.

**Figure 4:**
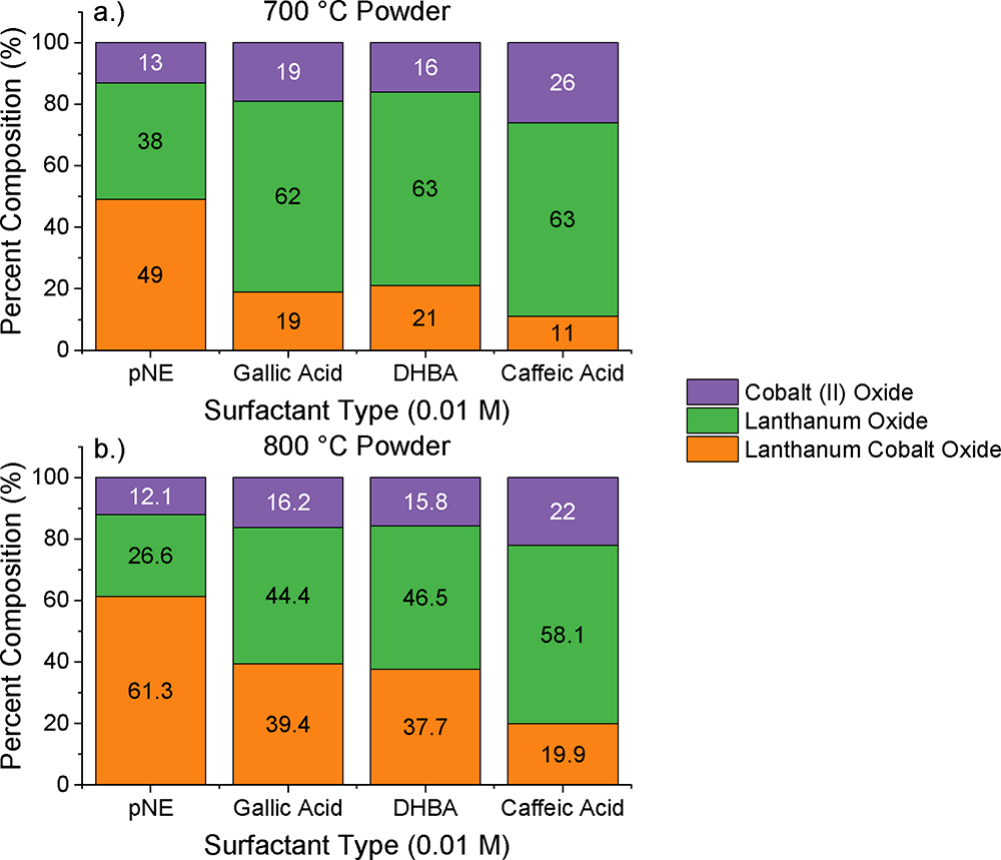
Percent phase development within the LCO powders synthesized using a surfactant molarity of 0.01 M, fired at (a) 700 and (b) 800 °C.

A similar study was completed for all catechol molecules, where the concentration of the chelating agent was varied when mixed into the precursor salt solutions. The 0.01 and 0.1 M solutions of each catechol were chosen to relate surfactant type and concentration to phase formation. LCO solution concentration was kept constant at 0.5 M, with a calcination temperature of 800 °C. Rietveld analysis of each powder from this study resulted in phase formation percentages, which are shown as comparative bar graphs in Figure 5. Analyzed XRD spectra of the 0.1 M used for this analysis can be found in Figure S1, with peaks associated with LCO marked. For all four tested molecules, increased surfactant concentration resulted in an increased phase-purity of LCO in the final powder. Of all surfactant types, pNE was found to be the best catechol molecule to properly chelate the desired ternary phase, having the highest percentage of LCO formed at both 0.01 and 0.01 M concentrations. At a concentration of 0.1 M, pNE formed the highest overall purity powder, where LCO was formed at ∼88.4%. It should be noted that the only recognized impurity for this sample was cobalt oxide. This may signify that a lanthanum deficiency during synthesis left excess cobalt unreacted, meaning that the phase purity could be higher. From these results, it was determined that pNE solutions (at a 0.1 M concentration) will be used as the chelating and surfactant in all further experiments in this current work.

**Figure 5:**
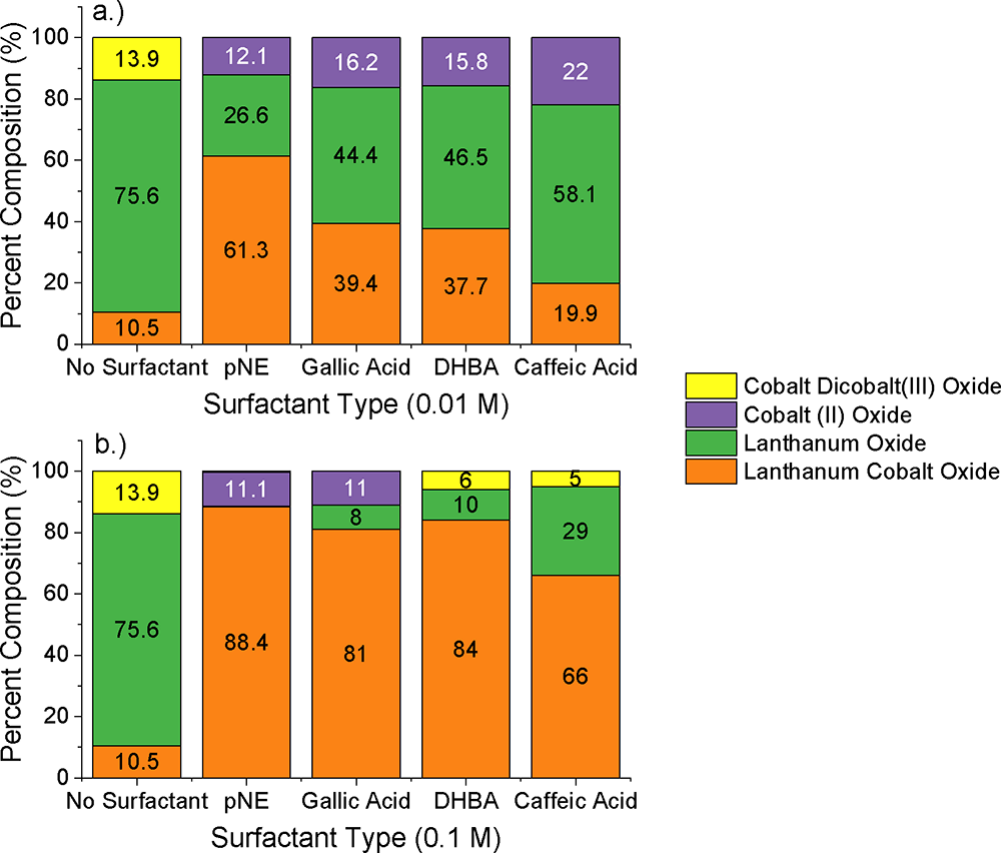
Percent phase development of LCO powders fired at 800 °C, created using surfactant molarities of both (a) 0.01 and (b) 0.1 M.

### 3.2 Effects of Deposition Time and Solution Molarity on LCO Nanostructure (AFM Study)

Before the implementation of the optimized nanoparticle solution chemistries, the effects of the precursor salt solution molarity and deposition time on the nanostructure were studied on polished, single-crystal YSZ substrates. The single crystal surface was initially scanned using noncontact mode over an area of 0.5 × 0.5 μm^2^ to characterize the initial baseline surface without any coating. Figure 6a shows a top-down planar view and a 3-dimensional topographical view of the AFM image of the baseline single crystal surface. Representative locations of the crystal were analyzed, and average roughness (*S*_a_), RMS roughness (*S*_q_), kurtosis (*S*_ku_), and surface area were calculated for the selected areas of the sample. The baseline showed an *S*_a_ of 40.86 pm, and an *S*_q_ of 50.76 pm, indicating that the samples were relatively flat with peaks in the picometer range, and the *S*_ku_ of 2.772 and very low surface area of 0.01004 μm^2^ confirmed the topography would not interfere with analysis of the nanoparticle decoration.

**Figure 6:**
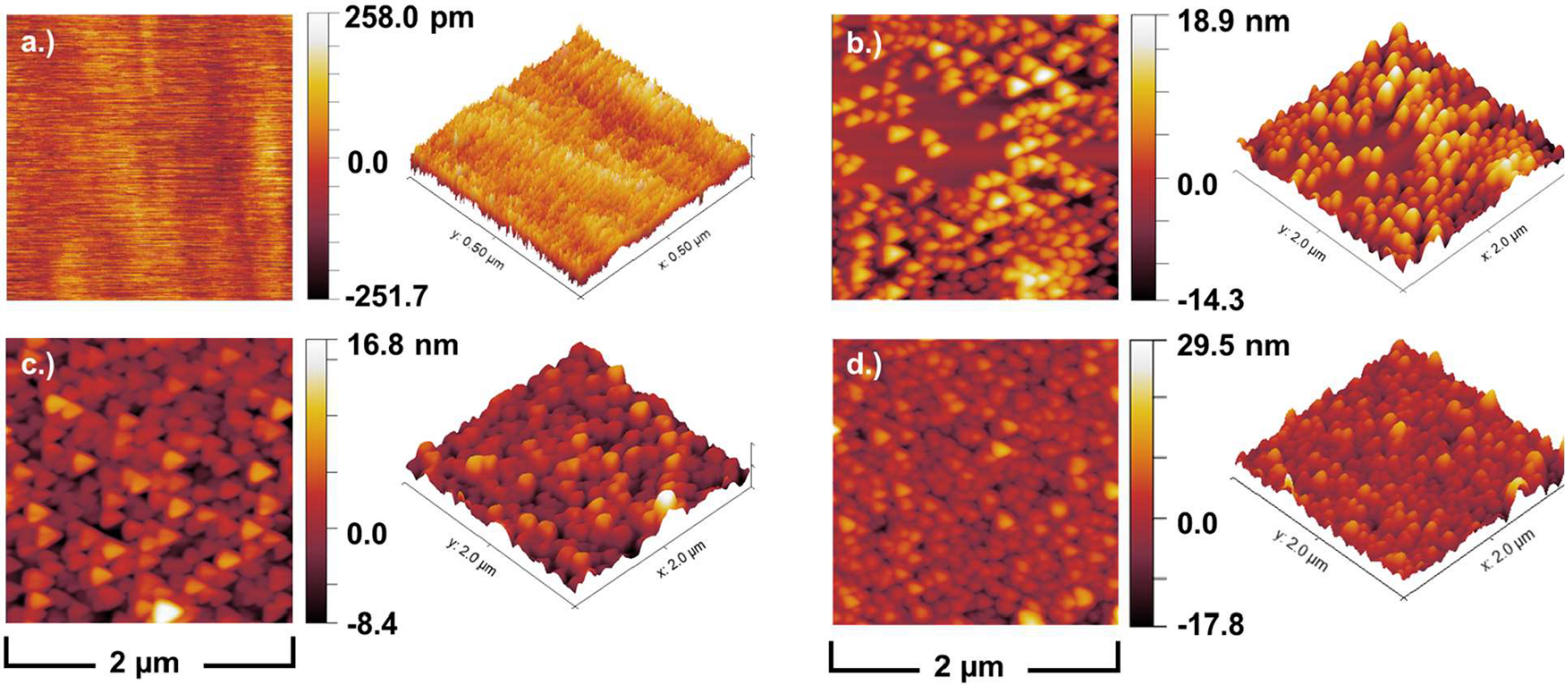
AFM maps of (a) a baseline YSZ substrate, 0.5 M nanoparticle depositions agitated for (b) 6 and (c) 12 h on single crystal YSZ, and (d) 0.1 M nanoparticle deposition agitated for 12 h.

Nanofilm deposition was then completed in a manner replicating the infiltration method for porous electrodes, as discussed in Section 2.2.1. Figure 6b,c shows a comparison between 2 × 2 μm^2^ representative LCO depositions on the surface with sample agitation times of 6 and 12 h, completed at a solution molarity of 0.5 M. Figure 6d shows a representative area where deposition conditions were 12 h deposition time and a molarity of 0.1 M, which enables determination of coating differences caused by variation of solution molarity when compared to Figure 6c. *S*_a_, *S*_q_, *S*_ku_, and surface area were also calculated across the selected areas, with a summary of the parameters in Table 1. All three samples showed a large, interconnected network of LCO particles with varying levels of coverage. Important distinctions were found when the LCO nitrate solution agitation times were varied. The roughness of the samples decreased with a longer deposition time, reflecting the increase in the nanoparticle packing density and nucleation sites. This increased loading was seen visually from the AFM maps. Differences were also seen related to the changes in the LCO solution molarity. Similarly to the deposition time, as molarity increased, roughness decreased, which indicates that a higher molarity solution will result in a denser coating and a smoother surface. This could be caused by an increase in nucleation sites during deposition, which contributes to the lower nanoparticle growth size during the growth step of infiltration. To improve charge transfer properties, a higher surface area is desired to increase active surface oxygen ion exchange sites throughout the cell, so a rougher sample, which correlates with a higher surface area, may be more desirable. In this regard, the best-performing deposition conditions were the 12 h, 0.1 M deposition, which had the highest surface area (4.1 μm^2^) and roughness (4.457 nm).

**Table 1:** Comparison of Roughness and Distribution of LCO Nanoparticles on YSZ Substrates

**deposition variables**	***S***_**a**_	***S***_**q**_	***S***_**ku**_	**surface area**
baseline sample	40.86 pm	50.76 pm	2.772	0.01004 μm^2^
6 h, 0.5 M	3.423 nm	4.457 nm	4.468	4.089 μm^2^
12 h, 0.5 M	2.466 nm	3.180 nm	4.441	4.044 μm^2^
12 h, 0.1 M	4.457 nm	5.461 nm	2.876	4.100 μm^2^

Rinsed samples, as described in Section 2.2.1, were next analyzed, with a focus on characterization of individualized particles. Figure 7 shows the distribution chart of particle size calculated from these samples using the equivalent disc radii. The average nanoparticle size was calculated to be ∼40 nm, with a normal particle size distribution around this average. The combined distribution and particle size information found that the pNE-enabled infiltration results in even nanoparticle formation and growth at low temperatures, thereby confirming that this method of infiltration can be utilized effectively for in situ SOEC coatings.

**Figure 7:**
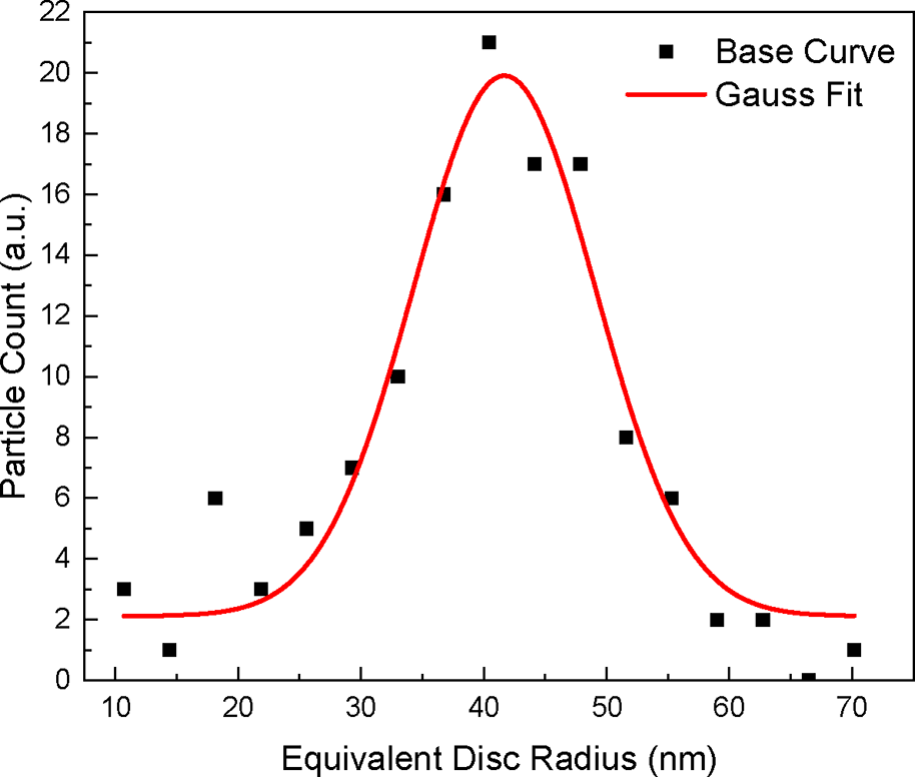
Graph of calculated equivalent disc radii of all mapped nanoparticles on a rinsed substrate, with a Gaussian fit to show normal distribution.

### 3.3 XPS Study of LCO nanocoated YSZ Substrates

To confirm the formation of the ternary LCO phase when deposited as a nanofilm coating and complement the prior XRD results, XPS was completed on the LCO film deposited on the single crystal YSZ. pNE and LCO solution concentrations were set at 0.1 and 0.5 M, as determined as optimal for XPS evaluation by AFM testing. Figure 8 shows the obtained lanthanum, cobalt, and oxygen XPS peaks from these samples. Figure 8a shows the La 3d region. Peak analysis determined that the photoemission line showed a satellite structure typical of the La^3+^ state, which is represented by the states 3d_5/2_ and 3d_3/2_ peaks. The peak spectra and positions agreed with those reported in the literature.^25,26^ The binding energy difference between the 5/2 and 3/2 states was found to be 16.8 eV, while the intrinsic separation difference of each doublet peak was determined to be ∼4 eV, with both values being consistent with prior studies on LCO.^27^

**Figure 8:**
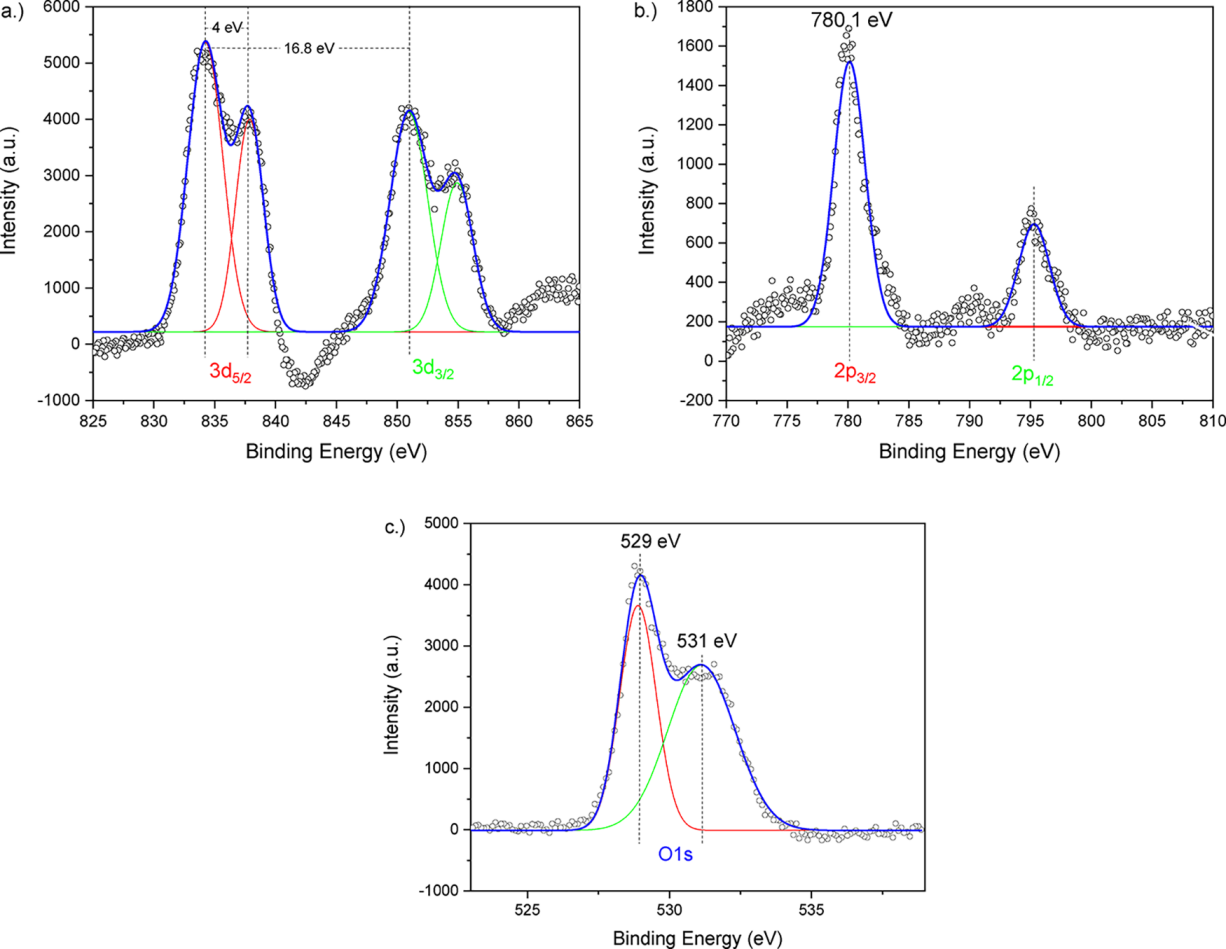
XPS spectra of (a) lanthanum, (b) cobalt, and (c) oxygen regions of LCO nanoparticle structures chelated by pNE on single crystal YSZ.

Analysis of the Co 2p region is shown in Figure 8b. The emission spectra corresponding to the 2p_3/2_ orbital exhibited a binding energy of 780.1 eV. This binding energy is characteristic of the Co^3+^ state. Previous authors indicated the possible presence of Co^2+^ formation,^28^ or cobalt hydroxide, at the surface level as the sample annealing temperature exceeded 700 °C.^26^ However, the narrow peak corresponding to the cobalt 2p_3/2_ state indicates the proper chemical environment of the single cobaltite phase. The shoulder typically seen in spectra with cobalt hydroxide phase impurities was not observed.^29^ These findings support the formation of a stable cobaltite structure.

Figure 8c depicts the O 1s region, which showed a doublet emission spectrum. The first peak, with a binding energy of ∼528 to 529 eV, was found to be indicative of lattice oxygen in the perovskite, while the second peak, with a binding energy of ∼531 eV, may be related to chemisorbed oxygen on the surface of LCO structures.^26^ The combination of these measurements validates that lanthanum and cobalt are correctly incorporated into the perovskite lattice, resulting in the desired LCO compound with no major observed impurities.

### 3.4 SEM Study of LCO Nanocoatings in Symmetrical LNO Cells, Pre- and Post-EIS Analysis

SEM characterization studies were completed to determine microstructural properties of the LNO symmetrical cell backbone and to characterize the in situ LCO nanoparticle deposition. Figure 9 shows a cross-sectional image of half of the baseline symmetrical cell, with dotted lines delineating the print regions. Each of these layers is easily distinguishable by the particle size difference. The GDC barrier layer densified almost entirely, enabled by the bimodal GDC particle mixture discussed in the experimental section. The barrier layer area showed a distinct lack of pores, a difference from the semiporous YSZ electrolyte. The mixed interlayer was seen to be less dense, with a larger LNO structure interspersed with nanosized GDC. Both the GDC barrier layer and the mixed interlayer were used to increase adhesion between the cell layers due to differences in the coefficient of thermal expansion.^30–32^ Use of the composite LNO-GDC layer also added a level of chemical and particle size similarity. No cracks or delaminations were seen across samples with a mixed interlayer, as opposed to cells where LNO bulk layers were printed directly on dense GDC. LCO-coated cell samples were imaged both before and after EIS testing. Figure 10 shows LNO symmetrical cells with and without LCO nanocoating before being run through the EIS treatment. The infiltrated sample shows a complex interconnected network of LCO on the surface of the cell as opposed to freestanding individual particles. The particle size agreed with that attained via AFM, but the nanocoating appeared far more connected than previously seen. This is thought to result from a higher level of interaction in the nanoporous area, resulting in longer and more complex polymer chains. The size of these structures lies in the ∼40 nm range, greater than that found via AFM. Though larger, the LCO layer maintained a submicron-level coating across the cell, which was a desirable result, as the perovskite LCO was chosen as a surface nanocoating to improve the transfer properties of LNO via the addition of a different conductivity mechanism with high surface area. Increased surface area should have the effect of increasing active sites, improving the surface oxygen exchange transfer reaction, and decreasing ASR throughout the cell. The cause of the particle size increase is likely related to the increased interactivity rate of the polymerization reaction in the nanoporous structure, causing larger and more complex structures to form, which can subsequently incorporate more ions from the solution.

**Figure 9:**
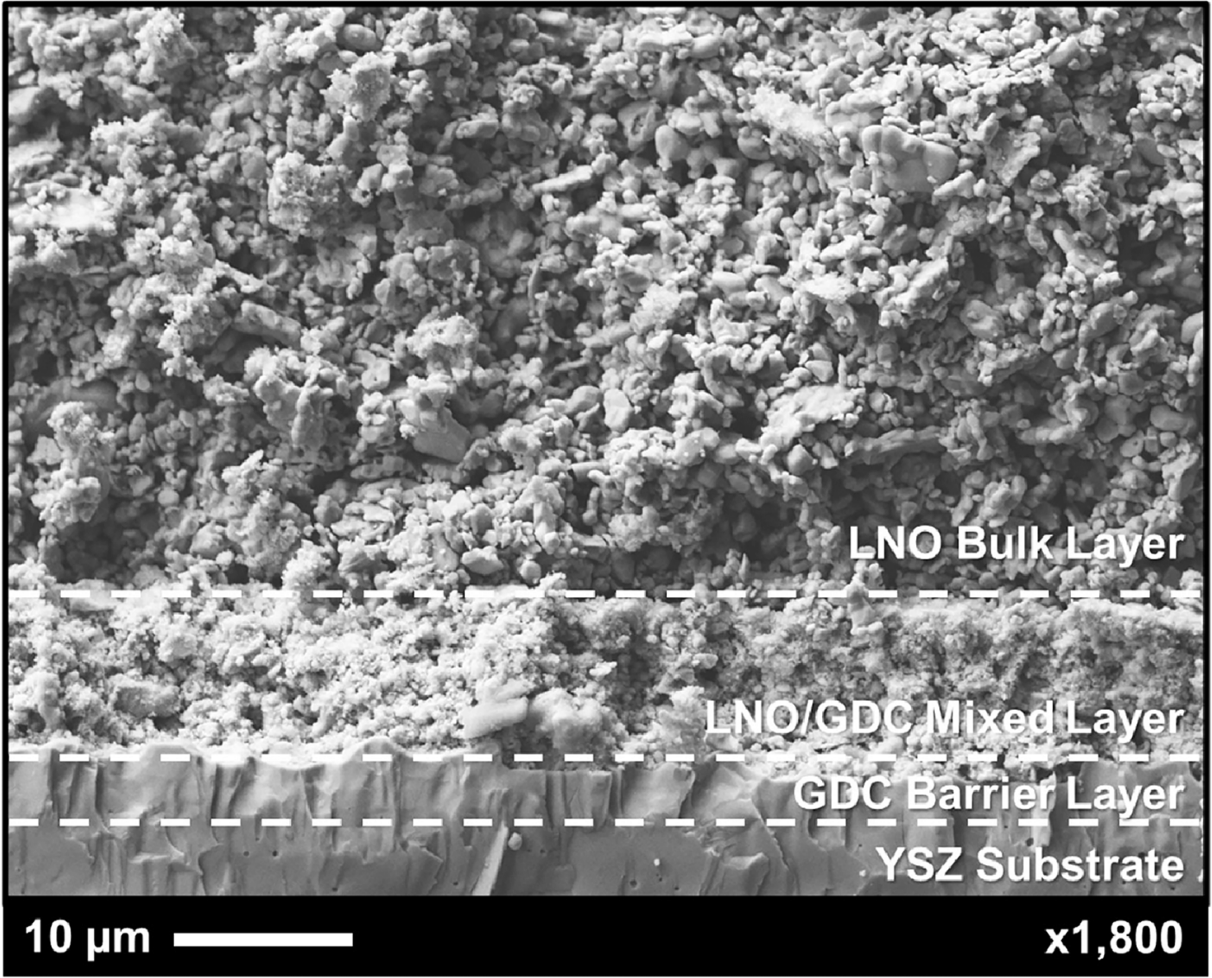
SEM micrograph of the cross-section of one side of the baseline LNO-based symmetrical cell used for the nanocatalyst infiltration experiments completed in this work.

**Figure 10:**
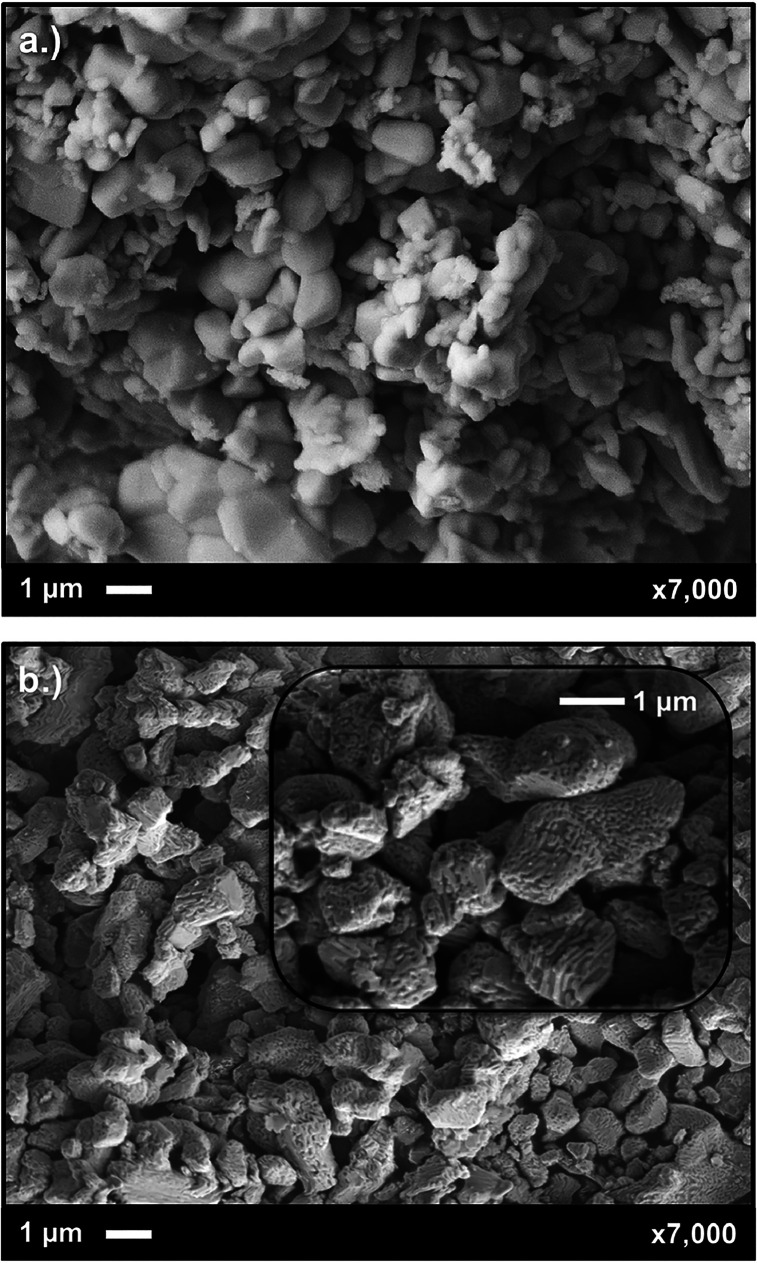
SEM micrographs of symmetrical LNO cells, both (a) with no nano-LCO coating and (b) infiltrated by LCO nanocoating.

Electrochemical performance (oxygen reduction reaction (ORR)) testing was completed on LCO-infiltrated SOEC samples at a range of temperatures, which is discussed in the following section. Samples from these tests were imaged to compare the changes in the nanostructure of the symmetrical cell layers and the LCO nanocoating. Figure 11 shows a comparison of LCO nanocoating in both pre- and post-EIS testing, with specific regions marked based on densification types. The pre-EIS coating (Figure 10a) showed the same complex interconnected nanocoating discussed earlier, with higher coverage of the LNO backbone and fewer pores. The post-EIS deposition (Figure 10b) showed signs of coating densification, with two distinct nanostructure types seen. A majority of the coated surfaces showed the nanocoating type denoted as Region A, which resembles a much denser pre-EIS deposition. This region showed a mixture of larger, interconnected particles with smaller pores. The second type, represented in Region B, showed completely interconnected nanoparticles with little to no pores between nanoparticles, forming what seems more like a grain boundary. This behavior is most likely caused by these areas having a denser LCO coating before EIS testing. Both coating types are a result of LCO particles sintering together and densifying, with the difference between the two likely stemming from the amount of LCO nanoparticle loading in the selected area. As LCO coverage increases, the densification of the coating over time increases. The tendency of LCO to shift in this way may indicate that a slightly lower loading of the cell would be more optimal, allowing nanostructures to better maintain individual structures by increased separation of the nanoparticles, thereby mitigating sintering mechanics.

**Figure 11:**
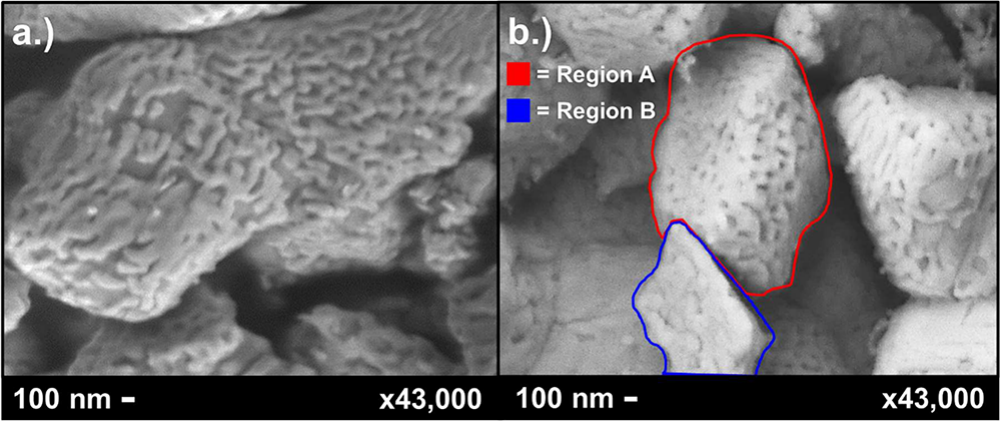
SEM micrographs of LCO-coated cell, both (a) before and (b) after EIS testing.

### 3.5 EIS of the LCO Infiltrated Half-Cells

The symmetrical cells were prepared, and EIS testing was completed to determine the impact of LCO nanocoating on the polarization resistance of the LNO electrode. The experimental variables tested were LCO infiltration times (6, 12, 24 h), LCO infiltration molarities (0.1, 0.5), and EIS testing temperature (600–800 °C). Cells were tested starting at 600 °C and then increased in 50 °C increments with ample hold time at each step to thermally equilibrate the system before testing. The prepared symmetrical LNO electrode cells resulted in microstructures or electrochemical properties that are not fully optimized, so all results from this study are compared to their baseline cell (the noninfiltrated LNO counterpart). All Nyquist diagrams were normalized to the area of the LNO backbone, halved to account for cell symmetry, and had their Ohmic resistances subtracted to present the electrode polarization resistance.

The Nyquist and Bode plots from the tested cells were analyzed. Figures 12 and 13 show comparisons of the baseline data and the 12 h LCO infiltrated cells (for the infiltration concentrations of 0.1 and 0.5 M) at an operating temperature of 700 and 800 °C, respectively. All samples with the nanocatalyst deposition showed improved electrochemical performance over the baseline sample. Figure 12 shows the Nyquist and Bode data for the 12 h infiltrations at 700 °C. While both infiltration concentrations improved against the baseline (0.4 Ω·cm^2^), the 0.1 M infiltrated cell’s polarization resistance of 0.152 Ω·cm^2^ outperformed the 0.5 M infiltration (0.28 Ω·cm^2^). This trend was shown across all three infiltration times performed at this temperature. Figure 13 shows the Nyquist and imaginary Bode data for the 12 h infiltrations tested at 800 °C. At this temperature, the 0.5 M infiltrated cell displayed a polarization resistance of 0.039 Ω·cm^2^, a reduction of ∼55%, and the 12 h deposition at 0.1 M resulted in a similar performance (0.042 Ω·cm^2^). This behavior indicates that the increase in temperature from 700 to 800 °C resulted in an increase in performance for the 0.5 M coating. This performance boost is thought to be a result of the coarsening of the microstructure (as shown in Figure 11), where at 800 °C the 0.5 M LCO nanocoating densified to reach a similar microstructure, and thus the triple-phase boundary area was similar to that of the 0.1 M coating. This coarsening (and densification) would then result in similar electrochemical performance for the two tested samples. All discussed impedance values from the Nyquist plots and those at other testing temperatures can be found in Table S1.

**Figure 12:**
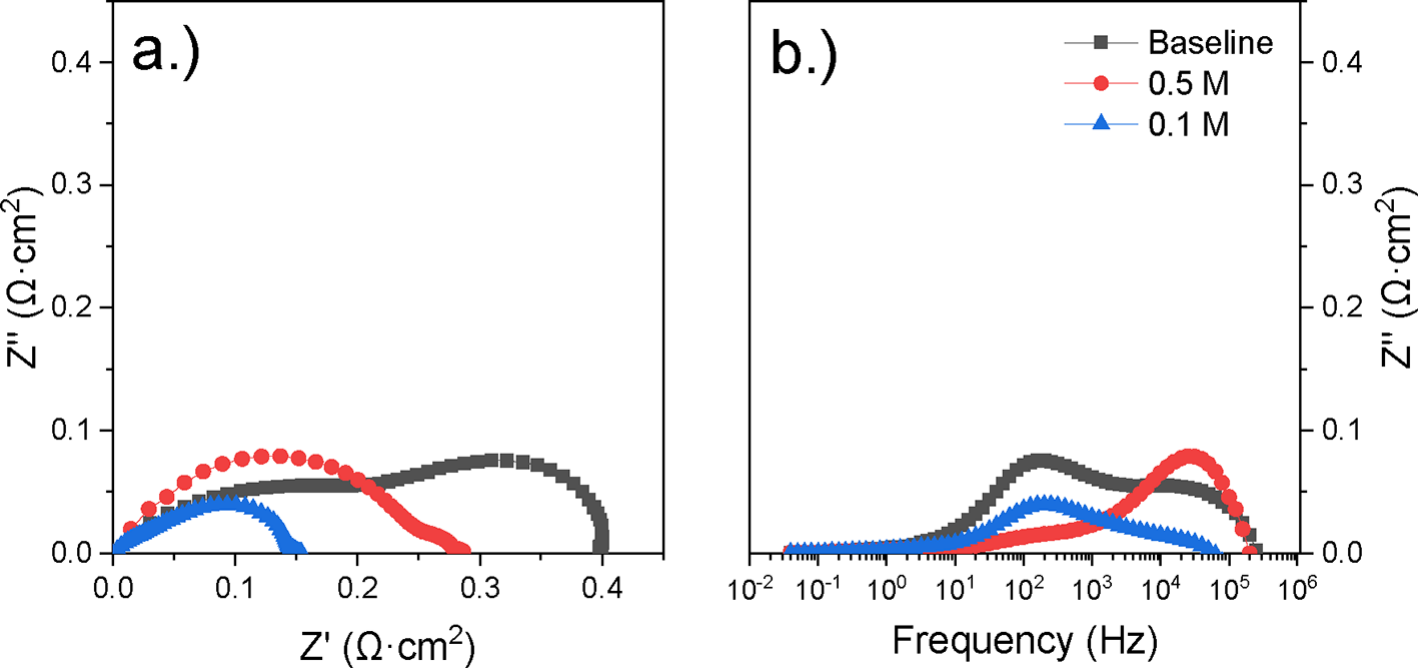
(a) Nyquist plot of baseline and 12 h, varied molarity LCO-infiltrated cells calcined at 700 °C, and (b) the corresponding imaginary Bode plots for each sample.

**Figure 13:**
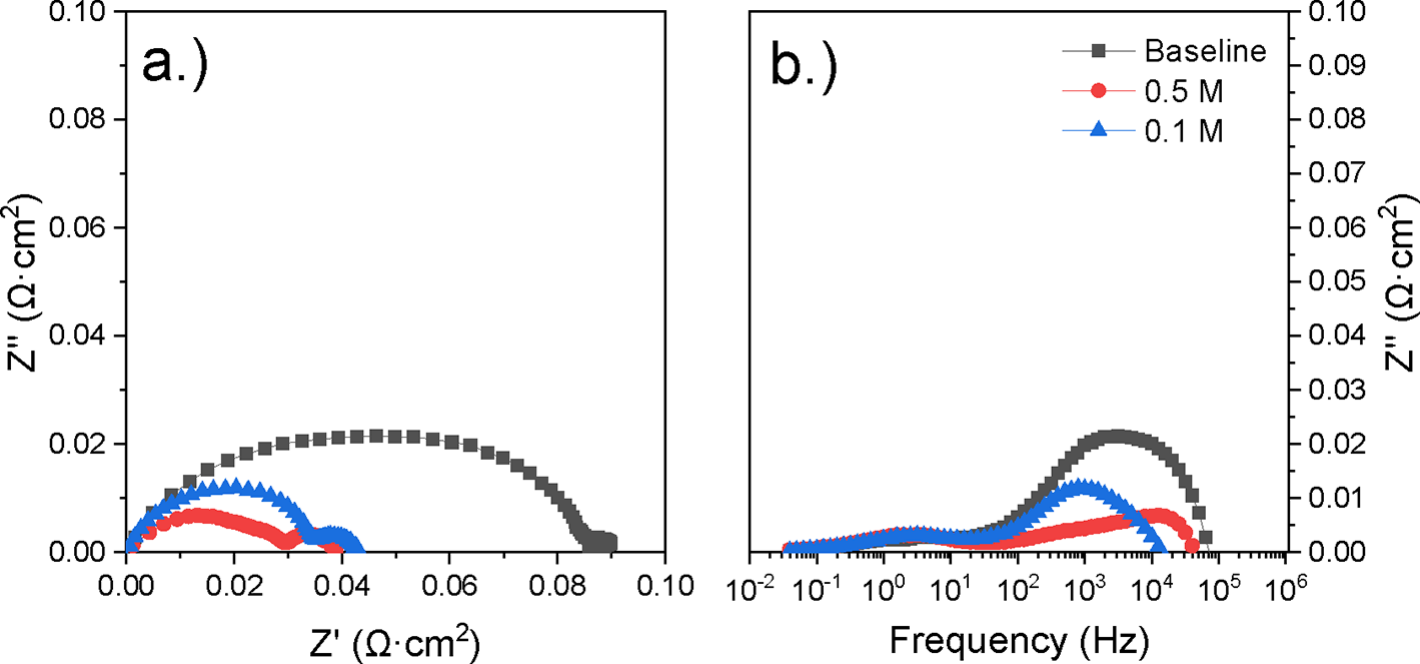
(a) Nyquist plot of baseline and 12 h, varied molarity LCO-infiltrated cells calcined at 800 °C, and (b) corresponding imaginary Bode plots for each sample.

The Bode plots of these samples were analyzed to determine the factors contributing to the electrochemical response. In the work from Yang et al., a model was developed for LSM-YSZ where the Bode data were deconvoluted to determine the impacts of microstructural and chemistry changes on the processes involved in the various adsorption, diffusion, reactions, and charge transfer mechanisms.^33^ For reference, this work found the adsorption reaction correlated to a peak between the 10^1^ and 10^2^ Hz regions, the diffusion of intermediate oxygen ions to have a peak appearing in the 10^2^ Hz region, and the charge transfer reaction to peak between the 10^^3^^–10^4^ Hz frequency range. The gas diffusion and bulk diffusion of oxygen vacancies processes were found to have overlapped peaks with R_ads_ and D_O_^–^_ad_, but both show low resistance contributions, meaning they are less likely to appear in an unseparated Bode frequency. The frequency ranges found for the constituent reactions and diffusion processes were used to compare the performance of the LCO-coated LNO cells. Analysis of cells across the tested temperature range found that the peaks correlated to the adsorption reaction shifted with increasing temperature from slightly before the 10^2^ Hz region to the 10^3^ Hz region. The peak magnitude of the *R*_ads_ peaks was halved for the 0.1 M samples and decreased by about 75% for the 0.5 M samples. A substantial change was also seen in the 10^5^ Hz range, correlated to the charge transfer reaction, where the LCO-infiltrated cells’ impedance showed a decrease of half or more compared to the baseline samples. These results align with the literature, as LNO used as an air electrode in an SOEC was shown to have high ASR as a result of its slow surface exchange reaction.^15,16,34^ Perovskite LCO’s role as a surface nanocoating was intended to improve the LNO backbone performance by adding and improving a different conduction mechanism, which analysis of the Bode plots seems to support.

The effect of the infiltration concentration on polarization resistance and activation energies was evaluated. The polarization resistances determined from the Nyquist plots were graphed as Arrhenius plots at 50 °C temperature increments. To show the overall trends of the infiltration molarities, all polarization resistance values were averaged across each tested set and compared to baseline cells, as shown in Figure 14. All infiltrated cells show major improvement against the baseline cells, with the 0.1 M samples having smaller impedance values across most of the lower temperature spectrum. The 0.5 M cells show a strong performance boost at 800 °C, surpassing the performance of the 0.1 M cells, whereas the baseline cells decrease in the level of improvement.

**Figure 14:**
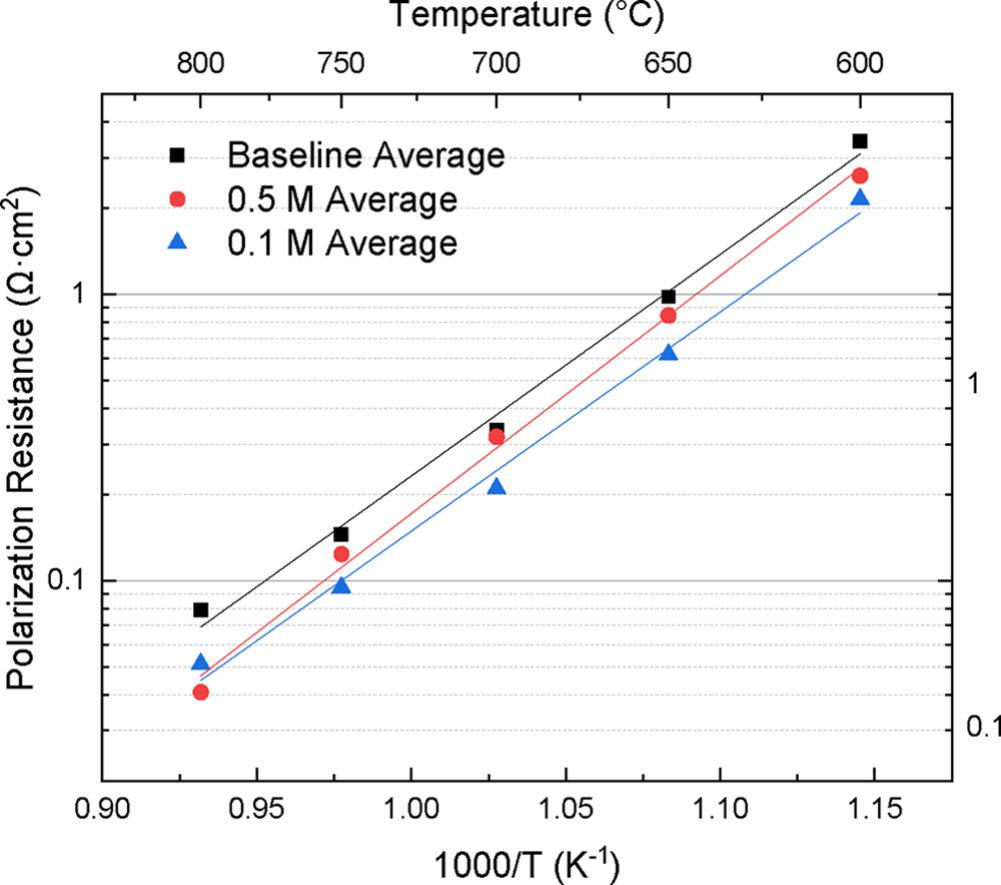
Arrhenius plot of polarization resistances comparing the effect of 0.1 and 0.5 M LCO concentrations on LNO cells against baseline, noninfiltrated cells.

This trend was more pronounced in the Arrhenius plots of the specific infiltration times, which are shown in Figure 15. For both chosen molarities, the 6 and 24 h infiltrations showed a mild boost in the improvement of the polarization resistances, while the 12 h depositions showed the best performance across all testing temperatures. This behavior could be explained by the underdeposition of nanoparticles in the 6 h infiltrations, leading to smaller changes in electrochemical behavior, and overdeposition in the 24 h infiltrations, causing nanopore blocking and oxygen starvation. As shown previously in the average data, the 0.5 M samples all showed a sharp increase in improvement at the 800 °C mark, becoming equal to or better than the best-performing 0.1 M sample. This change is thought to be a result of the densification of the nanocoating at the increased temperature, which may act to allow increased charge transfer through the interconnected nanocoating networks. This kind of densification of the nanoparticle coatings was confirmed in post-mortem SEM imaging, as discussed previously in Figure 11.

**Figure 15:**
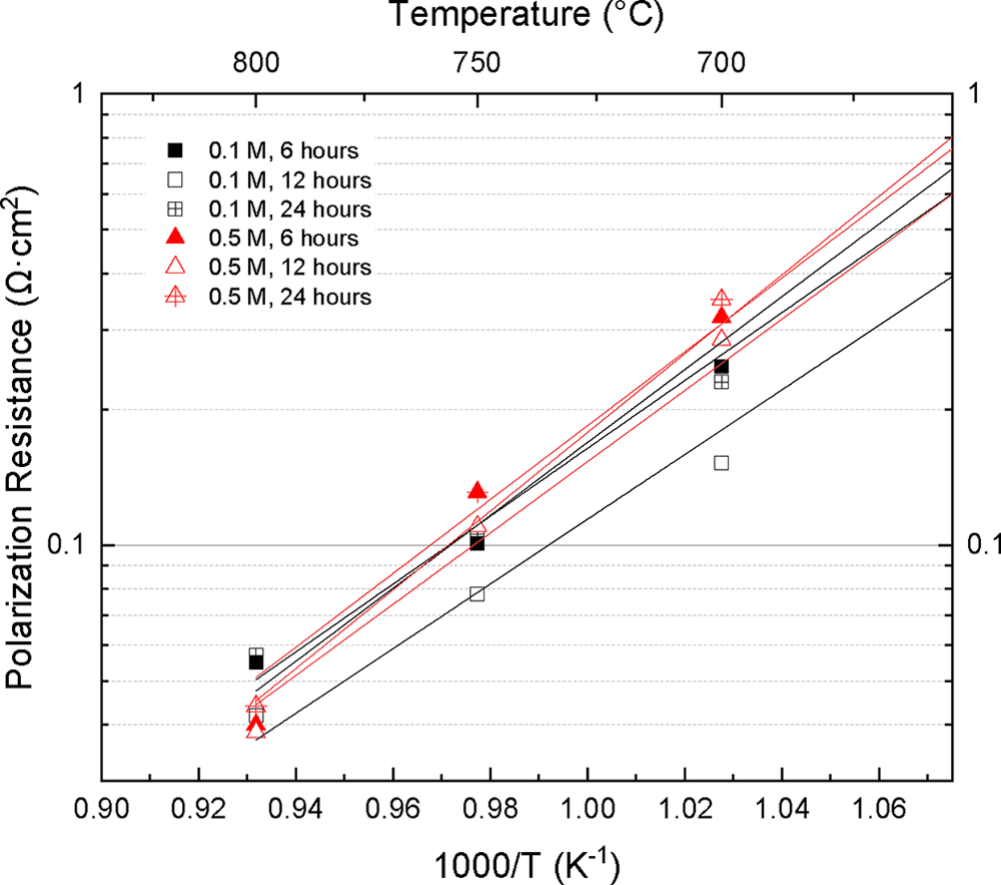
Arrhenius plot of LCO-infiltrated symmetrical cells at both 0.1 and 0.5 M concentrations, comparing deposition times of 6, 12, and 24 h.

Similar trends were also seen in the activation energies calculated from the Arrhenius diagrams, which are listed in Table S2 in the Supplementary Section. Both baseline and infiltrated samples had similar activation energies, ranging from 1.4 to 1.7 eV. Despite the narrower range, subtle trends were found in correlation with deposition characteristics. The 0.5 M samples showed activation energy values higher than those of the 0.1 M results, which showed a decrease of ∼0.135 eV across all samples. As mentioned previously, the deposition times seemed most impactful on the performance of the cell, with the lowest activation energy being the 12 h, 0.1 M deposition at 1.423 eV, a marked improvement from the baseline 1.605 eV, though interestingly the 6 h infiltration did not affect the activation energy, and the 24 h infiltration improved the performance by less than the 12 h, at ∼1.492 eV. This seems to align with the theory that the lower infiltration under-deposits, and the higher infiltration starts to overdeposit and decrease electrochemical performance. The 0.5 M infiltration at 12 h also improved against the baseline, at ∼1.567 eV, with both the 6 and 24 h trends showing a pattern similar to that of the 0.1 M infiltration. Results from this data seem to indicate that increased nanocoating of the cell may reduce the temperature sensitivity of the reaction slightly, though this may be within a margin of error due to the small Δ*E*_a_ between the samples.

### 3.6 ECR Testing to Determine Oxygen Surface Exchange Coefficient and Activation Energies

To better understand the kinetics of the oxygen evolution reaction (OER) in SOECs, the ECR method was completed both on baseline, fully LCO-coated, and nanodecorated LNO bars. This was completed to determine the impact of surface engineering on the oxygen surface exchange coefficient, termed the *k*-values of the samples. Evaluation of *k*-values is critical for analysis of SOC electrode materials, as the ORR (SOFC cathode) and OER (SOEC anode) occur by surface exchange, where the magnitude of k directly correlates to the catalytic activity of the surface. Larger *k*-values allow easier adsorption of O_2_ gas and incorporation as O^2–^ into the electrode lattice, and vice versa. Curve fitting is shown in Figure S2, was completed from the relaxation data of the baseline, the full LCO-coated, and the nanocoated LNO samples, then used to calculate *k*-values for each condition, compiled in Table 2. Particularly of note from these numbers is that the nanocoated material shows superior *k*-values across the full thermal range when compared to the fully coated sample. This shows that the nanoparticulate coating method is more beneficial to improve catalytic activity than the total surface coating method.

**Table 2:** Oxygen Exchange Coefficients of Baseline, Fully LCO-Coated, and LCO Nanodecorated LNO Bars, Tested across a Range of pO_2_ and a Temperature of 700 °C

**samples**	**partial pressure of oxygen (atm)**	***k*-values** (cm/s)
LNO	0.2–0.4	2.14 × 10^–5^
	0.4–0.6	2.55 × 10^–5^
	0.6–0.8	3.00 × 10^–5^
	0.8–1.0	4.28 × 10^–5^
LNO + full LCO	0.2–0.4	2.27 × 10^–5^
	0.4–0.6	2.64 × 10^–5^
	0.6–0.8	3.09 × 10^–5^
	0.8–1.0	4.91 × 10^–5^
LNO + nano-LCO	0.2–0.4	2.42 × 10^–5^
	0.4–0.6	2.96 × 10^–5^
	0.6–0.8	3.24 × 10^–5^
	0.8–1.0	6.13 × 10^–5^

Larger bulk disc samples were pressed and coated to compare only the baseline LNO to the full LCO-coated LNO. This was completed to analyze the improvement of *k*-values across a thermal range and determine activation energies of the surface oxygen exchange reaction, with the larger size of the pellets serving to exaggerate the trends present. As with bar samples, relaxation data were processed to calculate *k*-values for each pressure range. These values were then graphed as an Arrhenius plot, as shown in Figure 16. This diagram shows the evolution of the *k*-values at the tested temperatures and across a variety of pO_2_ ranges. These points were fit linearly to determine the activation energy of the oxygen surface exchange reaction. Table S3 contains a full list of both the *k*-values processed from these disc samples, and Table 3 shows the extrapolated activation energies. This plot shows the improvement of both the baseline LNO and the LNO + LCO-coated pellets across the full range of temperatures, which is the expected trend for this material. The *k*-values also show that for both coated and uncoated LNO, the oxygen exchange kinetics of the sample improve with increased partial pressures, correlating to the higher availability of oxygen to the system. Notably, the activation energy of the LNO + LCO sample was lowered by about 30% from the baseline LNO sample. This is a substantial decrease in the activation energy, demonstrating the effectiveness of LCO at improving the surface exchange reaction. The lower activation energies of the LNO+LCO sample also indicate that the LCO coating provides more stability for the cell against fluctuations in the operating temperature. There is a slight inflection at the 600 °C condition, which may indicate that there is an alteration with the charge transfer or absorption processes that together typically are Arrhenius in behavior. More research will need to be completed to better understand the causes behind the slight fluctuation at this data point. This understanding of the surface oxygen exchange properties is critical when designing and applying these kinds of complex nanomaterials to operational SOECs.

**Figure 16:**
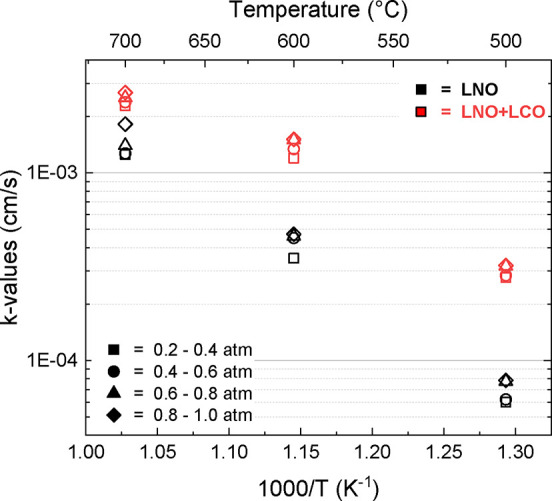
Development of the *k*-value for LNO and LCO-coated LNO as a function of temperature, measured across a range of pO_2_ values from 0.2 to 1.0 atm. The pO_2_ values are delineated by symbol, and the pellet type by color.

**Table 3:** Activation Energies of LNO and LNO + LCO Disc Pellet Samples across a Range of pO_2_ Changes

	**activation** energy (eV)	
**pO_2_ range (atm):**	**LNO**	LNO + LCO
0.2–0.4	0.986	0.690
0.4–0.6	0.986	0.698
0.6–0.8	0.942	0.680
0.8–1.0	1.020	0.697

## 4 Conclusions

A method to deposit a thin film of nano-LCO throughout a porous LNO-GDC electrode was achieved by using a two-step surfactant-enhanced solution impregnation process. This process permitted the controlled deposition of a ternary nanocatalyst composition in a single salt deposition, where typical aqueous infiltration methods take multiple deposition-drying steps to reach the desired loading levels for performance enhancement. The infiltration process utilized a catechol-structured organic molecule, which was first deposited regularly throughout the LNO microstructure, whereafter a salt solution containing La and Co ions in equal concentrations was impregnated into the structure. Multiple catechol molecules were tested due to structural differences in their hydroxyl and amine groups to allow for evaluation of their impact on the chelating capabilities and homogeneity of the resultant coating created when using the selected catechols.

Each of the tested surfactant molecules successfully chelated and formed a LaCoO_3_ perovskite phase at a temperature of 800 °C. Of the tested molecules, pNE permitted the highest phase development at 800 °C, with only a fine amount of CoO remaining unreacted. Even at lower temperatures, such as 700 °C, pNE was found to be twice as effective in comparison to other complexing agents. This difference may be due to differences in hydroxyl group spacings between the pNE molecule and the three other tested surfactants. Further research would need to be completed to correlate these molecular differences with the efficiency of nucleation and chelation mechanics of ceramic nanoparticles. LCO-coated YSZ samples coated with pNE were analyzed using XPS to compare the phase structure of the synthesized powder to the actual LCO nanoparticulate coating. Where powder studies indicated that phase impurities may exist, the nanocoating structure showed no sign of this, demonstrating the precision of the catechol-infiltration method when it was utilized with higher-order oxide structures.

Analysis of the physical characteristics of the LCO nanocoatings resulted in useful knowledge of how deposition conditions impact the density and surface area of the resultant coating. LCO solution infiltration time and molarity were adjusted as controlling factors to impact roughness and, consequently, the resultant surface area of the deposition. Packing density of the nanoparticles can be reduced utilizing either variable, but reducing either too much may result in an undersupply of La and Co ions, resulting in fewer nanoparticles and reducing the surface area for reactions in the other direction.

The LCO coating was predicted to improve the electrochemical properties of the cell by modifying the OER reaction to increase the surface ionic exchange rate of the LNO electrode. These benefits were confirmed by both EIS and ECR testing. The best performance was shown by a 0.5 M deposition at 12 h. Interestingly, though, for temperatures ≤750 °C, the 0.1 M deposition at 12 h showed better performance. The jump in the performance of the 0.5 M coating at 800 °C is attributable to an improvement in the charge transfer reaction. The cause of this could be densification of the coating, creating more pathways for O^2–^ diffusion. This densification would be less likely for lower molarity depositions, which have been shown to have less interconnected nanocoatings and, thus, could explain why this behavior is unique to the 0.5 M regime. Future work could focus on B-site substitutions with other compositions, such as Fe, Mn, and Ni, and the effects of these doped-LCO compositions’ stability and electrochemical performance in the LNO cell. The current research group has initiated work in this manner, focusing on the development of quaternary and high-entropy oxide compositions that have shown high resistivity to Cr poisoning and resistance to Sr segregation due to higher lattice distortion. Additionally, full-cell SOEC testing should be undertaken to determine the long-term impacts of LCO coating on the SOEC, full-cell improvement of the nanocoating, and how longer thermal hold periods impact LCO densification and potential phase development.

## Supplementary Material



## Notes

Supporting Information has been included, as referenced where relevant throughout the text. Figure S1 includes XRD diffractograms of the LCO powders with matched peaks labeled; Figure S2 includes collected and fitted data from the ECS testing of LNO, LNO+LCO, and nanocoated LCO bars. Table S1 includes normalized values of all polarization resistances of cells tested in the EIS section; Table S2 includes the activation energies of the cells processed from EIS data, and used to plot Arrhenius diagrams; Table S3 includes all oxygen exchange coefficients from the baseline LNO and LNO+LCO disc pellets across each tested pO_2_ and temperature range (PDF)

## Abbreviations

SOEC: solid oxide electrolysis cell
SOFC: solid oxide fuel cell
SOE: solid oxide electrolyzer
SOC: solid oxide cell
PEM: proton exchange membrane
LNO: lanthanum nickelate
LCO: lanthanum cobaltite
GDC: gadolinium-doped ceria
YSZ: yttrium-stabilized zirconia
pNE: polymerized norepinephrine
DHBA: dihydroxy benzoic acid
DOPA: dihydroxyphenylalanine
LSCF: lanthanum strontium cobalt ferrite
LSM: lanthanum strontium manganite
NNO: neodymium nickelate
PNO: praseodymium nickelate
R-P: Ruddlesden–Popper
CVD: chemical vapor deposition
ALD: atomic layer deposition
PLD: pulsed laser deposition
XRD: X-ray diffractometry
BET: Brunauer Emmett and Teller
XPS: X-ray photoelectron spectroscopy
AFM: atomic force microscopy
RMS: root-mean-square
SEM: scanning electron microscopy
ASR: area-specific resistance
EIS: electrochemical impedance spectroscopy
ECR: electrical conductivity relaxation
ORR: oxygen redux reaction
OER: oxygen evolution reaction
