# Supplementary material for: Optimization of La2NiO4+δ Electrolysis Cell Oxygen Electrode through Surfactant-Enabled LaCoO3±δ Nanocatalyst Deposition
Source: ACS Omega. 2025 Oct 29;10(44):53364–77. doi: 10.1021/acsomega.5c08373 (PMC12613117; doi:10.1021/acsomega.5c08373)
Supplement: Supplementary file 1 [file ao5c08373_si_001.pdf]

# Optimization of $La_2NiO_{4+\delta}$ Electrolysis Cell Oxygen Electrode Through Surfactant-Enabled $LaCoO_{3\pm\delta}$ Nano-Catalyst Deposition

Cole Klemstine[1], Javier Mena[1], Wenyuan Li[2], Awa Kalu[2], Xingbo Liu[1], Yu Zhong[3], Edward M. Sabolsky[1*]

[1]Department of Mechanical, Materials and Aerospace Engineering, West Virginia University, Morgantown, WV 26506, USA
[2]Department of Chemical and Biomedical Engineering, West Virginia University, Morgantown, WV 26506, USA
[3]Department of Materials Science and Engineering, Worcester Polytechnic Institute, Worcester, MA 01609, USA

*Corresponding author: Ed.Sabolsky@mail.wvu.edu

**Key words**: Lanthanum cobalt oxide (LCO), Symmetrical lanthanum nickel oxide (LNO) cells, Electrochemical performance, Nanocoatings, Electrochemical impedance spectroscopy (EIS).

**Abstract**

Lanthanum nickelate (LNO) has shown promise as a Cr-resistant air electrode material for SOECs but has suboptimal surface oxygen exchange properties. Nanocoating of the LNO surface with lanthanum cobaltite (LCO) was chosen to improve cell performance as a surface oxygen-conductor. The work focused on implementation of a two-step nano-LCO film deposition utilizing catechol molecules in a porous LNO electrode. The subgoals of the work were to maintain nanosized LCO particles/grains to increase active surface area, and to control the regularity/homogeneity of the coating across the microstructure. To achieve these goals, a novel surfactant-enhanced liquid infiltration method was utilized, where nucleation sites were spread across the electrode structure to control the location and size of LCO particles. Various catechol surfactant compositions were evaluated for their ability to control the kinetics of nanoparticle deposition and homogeneity of the coating. Chelated LCO was characterized by X-ray Diffraction (XRD), which found a substantial improvement in LCO formation with surfactant addition and determined polymerized norepinephrine to be the best-performing surfactant, with 88.4% pure LCO formed at low temperature. X-ray Photoelectron Spectroscopy (XPS) confirmed LCO lattice formation on nanostructures formed by two-step infiltration process, showing no impurities and a stable perovskite structure. Deposition kinetics were analyzed using Atomic Force Microscopy (AFM), correlating infiltration times and solution molarity to nanoparticle size and distribution, the results of which were confirmed in symmetrical cell samples by Scanning Electron Microscopy (SEM). Electrochemical Impedance Spectroscopy (EIS) testing demonstrated substantial improvements in polarization resistance, where nanocoating reduced impedance by ~55% to 0.152 $\Omega\cdot cm^2$ at 700 °C and 0.039 $\Omega\cdot cm^2$ at 800 °C. Electrical Conductivity Relaxation (ECR) at this temperature confirmed improved surface-oxygen exchange coefficient of the LCO+LNO heterostructure predicted by Bode data from EIS, alongside a reduction in activation energy by about 30%.

## Supplemental Figures

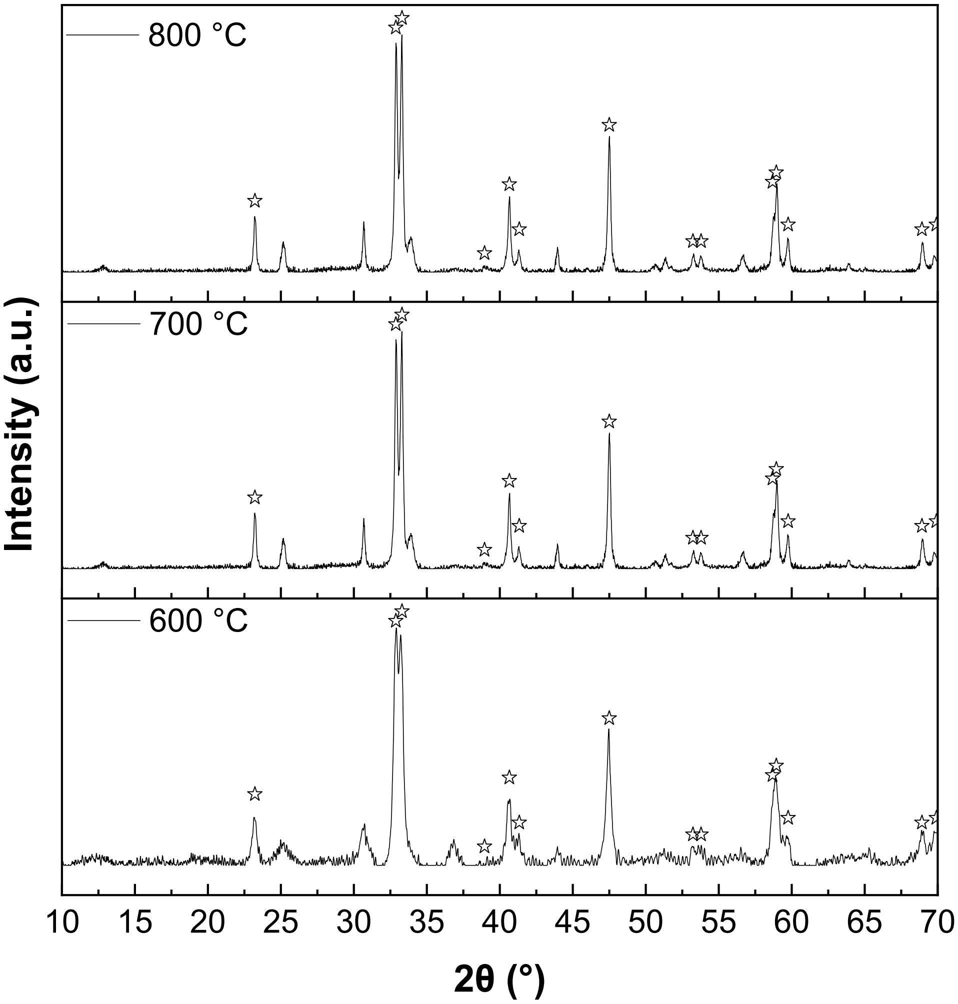


**Figure S1:** XRD diffractrogram of LCO powder synthesized at 600, 700, and 800 °C using pNE as a surfactant at a concentration of 0.1 M.

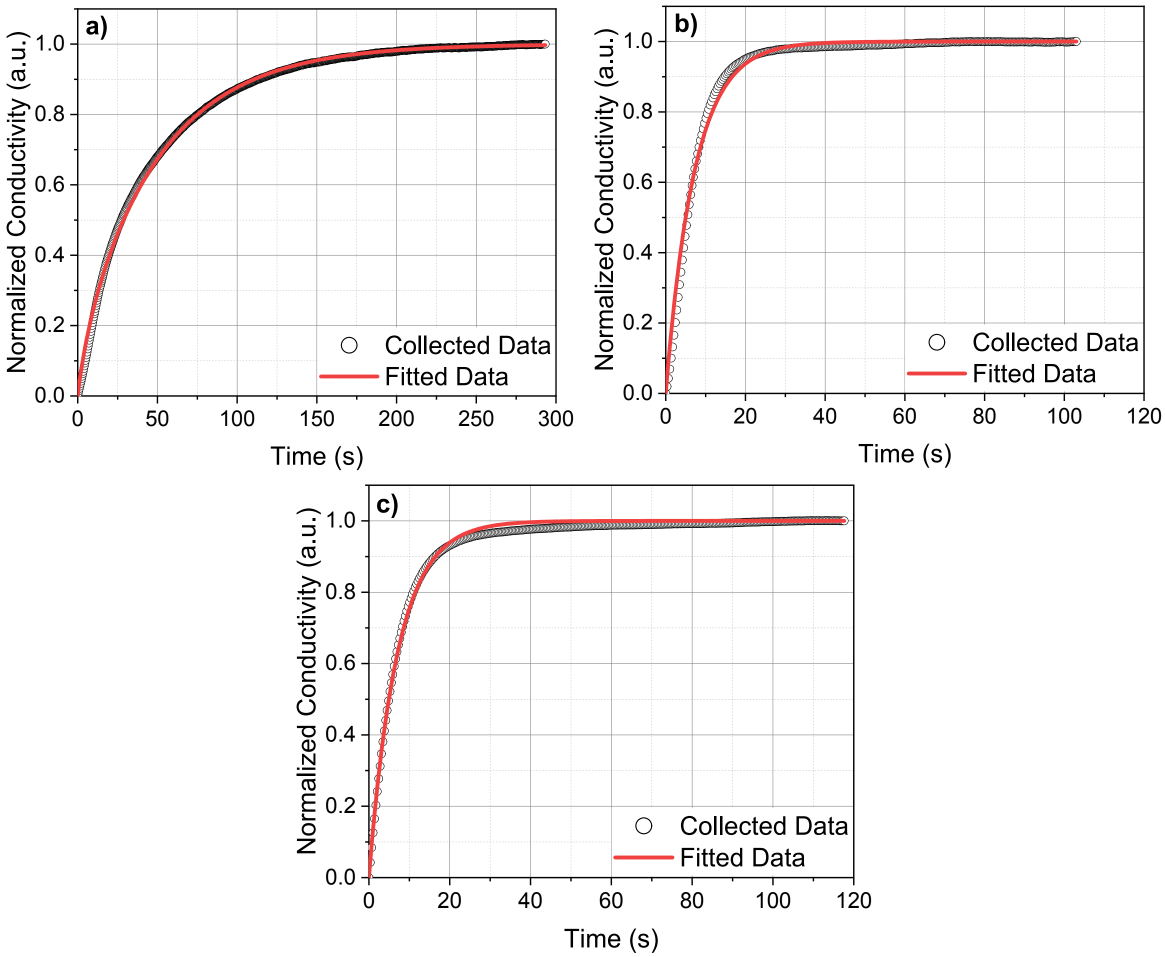


**Figure S2:** Fitting of the k-values of a) baseline LNO compared to b) LNO+LCO, and c) nanocoated LNO tested at a temperature of 700 °C and a partial pressure of oxygen at 0.6-0.8 atm

## Supplemental Tables

**Table S1:** Polarization resistances of cells tested in EIS, normalized to area of one electrode ($\Omega \cdot cm^2$)

| Temperature: | Baseline | 0.1 M Samples | | | 0.5 M Samples | | |
|---|---|---|---|---|---|---|---|
| | | 6-hour | 12-hour | 24-hour | 6-hour | 12-hour | 24-hour |
| 600 °C | 3.028 | 2.75 | 1.45 | 2.25 | 3.25 | 1.95 | 2.58 |
| 650 °C | 0.991 | 0.78 | 0.41 | 0.66 | 0.90 | 0.72 | 0.91 |
| 700 °C | 0.351 | 0.249 | 0.152 | 0.230 | 0.320 | 0.285 | 0.347 |
| 750 °C | 0.144 | 0.101 | 0.078 | 0.106 | 0.131 | 0.110 | 0.131 |
| 800 °C | 0.086 | 0.055 | 0.042 | 0.057 | 0.040 | 0.039 | 0.044 |

**Table S2:** Activation energies, measured in cells tested by EIS (eV)

| Baseline | 0.1 M Samples | | | 0.5 M Samples | | |
|---|---|---|---|---|---|---|
| | 6-hour | 12-hour | 24-hour | 6-hour | 12-hour | 24-hour |
| 1.605 | 1.603 | 1.423 | 1.492 | 1.731 | 1.567 | 1.624 |

**Table S3:** Oxygen exchange coefficients of baseline LNO and fully coated LNO+LCO disc pellets, tested across a range of $pO_2$ at and temperatures

| Samples | Temperature (°C) | Partial pressure of oxygen (atm) | k-values (cm/s) |
|---|---|---|---|
| LNO Disc Pellet | 500 °C | 0.2 – 0.4 | $6.01\times10^{-5}$ |
| | | 0.4 – 0.6 | $6.21\times10^{-5}$ |
| | | 0.6 – 0.8 | $7.75\times10^{-5}$ |
| | | 0.8 – 1.0 | $7.85\times10^{-5}$ |
| | 600 °C | 0.2 – 0.4 | $3.51\times10^{-4}$ |
| | | 0.4 – 0.6 | $4.50\times10^{-4}$ |
| | | 0.6 – 0.8 | $4.60\times10^{-4}$ |
| | | 0.8 – 1.0 | $4.72\times10^{-4}$ |
| | 700 °C | 0.2 – 0.4 | $1.25\times10^{-3}$ |
| | | 0.4 – 0.6 | $1.27\times10^{-3}$ |
| | | 0.6 – 0.8 | $1.40\times10^{-3}$ |
| | | 0.8 – 1.0 | $1.82\times10^{-3}$ |
| LNO+LCO Disc Pellet | 500 °C | 0.2 – 0.4 | $2.77\times10^{-4}$ |
| | | 0.4 – 0.6 | $2.84\times10^{-4}$ |
| | | 0.6 – 0.8 | $3.19\times10^{-4}$ |
| | | 0.8 – 1.0 | $3.21\times10^{-4}$ |
| | 600 °C | 0.2 – 0.4 | $1.20\times10^{-3}$ |
| | | 0.4 – 0.6 | $1.34\times103^{5}$ |
| | | 0.6 – 0.8 | $1.5\times10^{-3}$ |
| | | 0.8 – 1.0 | $1.51\times10^{-3}$ |
| | 700 °C | 0.2 – 0.4 | $2.28\times10^{-3}$ |
| | | 0.4 – 0.6 | $2.38\times10^{-3}$ |
| | | 0.6 – 0.8 | $2.52\times10^{-3}$ |
| | | 0.8 – 1.0 | $2.68\times10^{-3}$ |
